# Extracellular matrix hydrogel derived from decellularized tissues enables endodermal organoid culture

**DOI:** 10.1038/s41467-019-13605-4

**Published:** 2019-12-11

**Authors:** Giovanni Giuseppe Giobbe, Claire Crowley, Camilla Luni, Sara Campinoti, Moustafa Khedr, Kai Kretzschmar, Martina Maria De Santis, Elisa Zambaiti, Federica Michielin, Laween Meran, Qianjiang Hu, Gijs van Son, Luca Urbani, Anna Manfredi, Monica Giomo, Simon Eaton, Davide Cacchiarelli, Vivian S. W. Li, Hans Clevers, Paola Bonfanti, Nicola Elvassore, Paolo De Coppi

**Affiliations:** 1 Stem Cell and Regenerative Medicine Section, University College London GOS Institute of Child Health, London, UK; 2 Shanghai Institute for Advanced Immunochemical Studies (SIAIS), ShanghaiTech University, Shanghai, China; 3 Epithelial Stem Cell Biology & Regenerative Medicine Laboratory, the Francis Crick Institute, London, UK; 4 Oncode Institute, Hubrecht Institute, Royal Netherlands Academy of Arts and Sciences (KNAW) and University Medical Center (UMC) Utrecht, Utrecht, Netherlands; 5 Stem Cell and Cancer Biology Lab, the Francis Crick Institute, London, UK; 6 Telethon Institute of Genetics and Medicine (TIGEM), Pozzuoli, Italy; 7 Veneto Institute of Molecular Medicine & Dept. of Industrial Engineering, University of Padova, Padova, Italy; 8 Princess Máxima Center (PMC) for Pediatric Oncology, Utrecht, Netherlands; 9 Specialist Neonatal and Paediatric Surgery Unit, Great Ormond Street Hospital, London, UK

**Keywords:** Tissues, Extracellular matrix, Intestinal stem cells, Intestinal stem cells

## Abstract

Organoids have extensive therapeutic potential and are increasingly opening up new avenues within regenerative medicine. However, their clinical application is greatly limited by the lack of effective GMP-compliant systems for organoid expansion in culture. Here, we envisage that the use of extracellular matrix (ECM) hydrogels derived from decellularized tissues (DT) can provide an environment capable of directing cell growth. These gels possess the biochemical signature of tissue-specific ECM and have the potential for clinical translation. Gels from decellularized porcine small intestine (SI) mucosa/submucosa enable formation and growth of endoderm-derived human organoids, such as gastric, hepatic, pancreatic, and SI. ECM gels can be used as a tool for direct human organoid derivation, for cell growth with a stable transcriptomic signature, and for in vivo organoid delivery. The development of these ECM-derived hydrogels opens up the potential for human organoids to be used clinically.

## Abstract (web summary)

Organoid cultures have been developed from multiple tissues, opening new possibilities for regenerative medicine. Here the authors demonstrate the derivation of GMP-compliant hydrogels from decellularized porcine small intestine which support formation and growth of human gastric, liver, pancreatic and small intestinal organoids.

## Introduction

Organoids are three-dimensional multicellular constructs with a unique ability of both maintaining their stemness throughout the unlimited expansion, and functionally differentiating to mature phenotypes^1^. As a consequence, organoids are a promising cell source for tissue regeneration, tissue repair, and could be applied as a therapeutic tool for various disease models. Mouse organoids have been derived from various tissue types and extensively investigated both in vitro^2,3^ and in vivo^4^. Human organoids from different endodermal tissues have been extensively investigated in vitro^5,6^. Fewer studies have reported the investigation of human organoids for in vivo applications. Still, these models hold great promise for regenerative medicine^7,8^.

Organoids are commonly cultured in 3D hydrogel systems, which are highly hydrated polymer networks. However, to apply these culture systems in a clinical environment, various limitations must be overcome. One constraint relates to the ability to expand these organoids in conditions that are GMP-compliant. Most organoids are generated by the simple expansion of stem cells in 3D structures of extracellular matrix (ECM)-derived proteins. Mouse tumor matrices with poorly defined environmental signaling, such as Engelbreth-Holm-Swarm (EHS) sarcoma, have often been used for organoid expansion in vitro. Attempts to produce artificial matrices that could overcome these limitations for clinical translation have so far shown encouraging results in mouse^9^ and, recently, in human organoids^10^. However, it is a real challenge to define all the relevant information necessary to instruct specific tissue remodeling and regeneration. This is also related to the partial information in the literature about the biochemical signature of tissue-specific ECM^11,12^. As a consequence, synthetic matrices can only partially reproduce some of the native ECM features which commonly include adhesion signals and proteo-cleavable structures.

We envisage that the use of naturally derived materials from decellularized tissues (DT) could be compatible for clinical translation. Indeed, DT have been already used clinically, for example as cardiac valve substitutions and for the patch repair of large surgical defects^13,14^. Additionally, cell-laden DT efficiently promote in vivo tissue regeneration as demonstrated both by pre-clinical data and experimental human transplantation. These processes suggest that ECM not only provides a structural support, but also delivers biochemical signals that are fundamental to assisting the regeneration process^15^. These environmental cues are not limited to ECM tissue-specific proteins, but also include soluble factors absorbed within the ECM protein network.

Hydrogels derived from DT potentially have the advantage of providing the cells with all the information they need for their growth and expansion, while also being GMP-compliant^16^. Herein we explore the capability of a clinically compliant ECM gel to host formation and growth of endoderm-derived organoids.

To drive this hypothesis, we use decellularized porcine SI mucosa/submucosa^17^ to obtain a clinically compatible ECM self-gelling hydrogel. We demonstrate that intestinal ECM-derived gels have similar physiological range and mechanical properties to commercially available gels. Of relevance, they have the proteomic signature of endoderm tissue with specific enrichment of key ECM proteins relevant to organoid formation. We demonstrate formation and growth of endoderm-derived human organoids including small intestine (SI), liver, stomach and pancreas. Finally, ECM-derived gels support in vivo organoids growth. We envisage that ECM-derived hydrogels could be used in the future for organoid transplantation and bioprinting in clinically relevant environments^18^.

## Results

### Gelation and characterization of ECM-derived intestinal gel

**Fig. 1:**
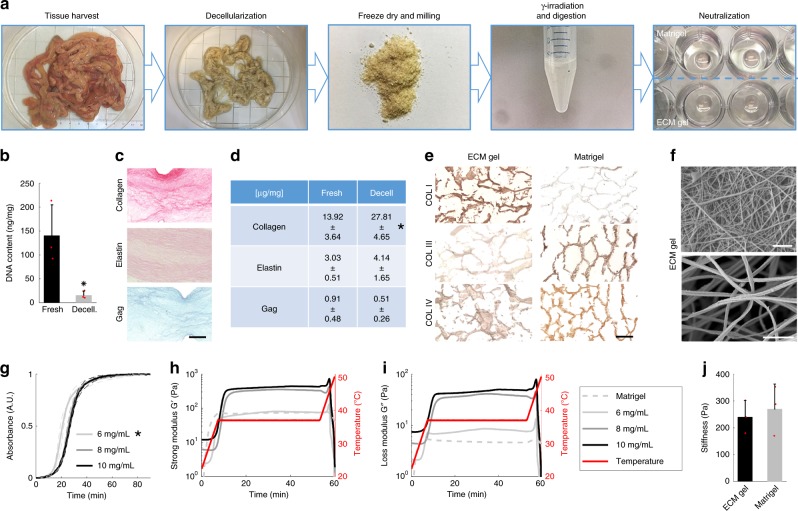
Extracellular matrix hydrogel characterization. **a** The gelation preparation protocol consists of decellularization of the SI mucosa/submucosa, freeze-drying process, milling into a fine powder, gamma-irradiating and digesting the powder in pepsin and HCl for 72 h, and neutralization to a physiological pH, salinity and temperature. **b** DNA quantification in fresh (immediately after organ harvest) and decellularized piglet mucosa. Mean ± S.D. (*n* = 3 small intestines). Two-sided *t*-test *P < 0.05. Asterisk denotes significance. Red dots show individual data points. **c** Histological sections of fixed ECM gel drops stained with Picrosirius Red, Verhoeff’s and Alcian Blue for collagen, elastin and glycosaminoglycans, respectively. Scale bar 200 μm. **d** Quantification of ECM proteins: collagen, elastin and GAG. Mean ± S.D. (*n* = 3 gel batches). Two-sided *t*-test *P < 0.05. **e** Analysis of the collagen types in ECM gel and Matrigel by staining for collagen I, III, and IV. Scale bar 100 μm. **f** Scanning electron microscopy (SEM) images of the ECM gel displaying the interconnected fibrous network. Scale bars 1 µm. **g** Spectrophotometry used to assess the turbidity of the samples during gelation. Mean ± S.D. (*n* = 2 gel batches). Two-sided *t*-test *P < 0.05 of T_lag_. **h**, **i** Oscillatory rheology provides a rheological profile of various concentrations of the ECM gel and Matrigel, for both **h** storage modulus and **i** loss modulus. **j** Elastic modulus measured by nanoindentation of 6 mg/mL ECM gel vs. Matrigel in 30 µL drops. Mean ± S.D. (*n* = 3 gel droplets). Two-sided *t*-test *P < 0.05.

To optimize a GMP-compatible process for ECM gel, we designed a 5 step protocol which includes (i) tissue harvesting; (ii) decellularization; (iii) freeze dry and milling; (iv) gamma-irradiation and digestion; and (v) neutralization based on modification of previously reported protocols^19–21^ (Fig. 1a, Supplementary Fig. 1a). One cycle of the detergent-enzymatic treatment (DET) facilitated nuclei removal and significant DNA decrease (Fig. 1b) in the porcine intestinal scaffold. This short protocol minimized morphological tissue alteration compared to other decellularization protocols, as previously reported^21^ (Supplementary Fig. 1b).

We first verified that ECM powder derived from porcine intestinal tissue successfully formed a hydrogel when following a gelation protocol. ECM powder was digested in pepsin and HCl, re-equilibrated to neutral pH and exposed to physiological temperature. The SI ECM decellularization and gelation efficiently preserved the relevant ECM components including collagens, elastin and still contained glycosaminoglycans (Fig. 1c, d). When compared to standard 3D culture systems, such as Matrigel, collagen I, III, and IV showed at least comparable signals (Fig. 1e). Solubilization of ECM by pepsin digestion was performed to preserve the ultrastructure of the collagen fibers, based on the fact that pepsin cleaves collagens in locations where the three alpha-chains are not interacting to form a stable triple-helical structure^22^. To further investigate the structure of the ECM hydrogels, scanning electron microscopy (ESM) was performed and showed the detailed interwoven network of collagen fibers (Fig. 1f).

Rheological and mechanical properties of 3D environment are extremely relevant to organoid culture. By spectrophotometry we assessed the turbidimetric gelation kinetics of the hydrogels (Fig. 1g). All the analyzed ECM hydrogel concentrations (6, 8 and 10 mg/mL) formed a sigmoidal curve indicating gelation, reaching the 90% of gelation in ~30 min for all three conditions. *T*_lag_ time was significantly lower in ECM gels at 6 mg/mL concentration, compared to 8 and 10 mg/mL gels (Supplementary Table 1). However, the ECM powder digested solution (pre-gel) needed to be freshly prepared and kept at +4 °C, or stored at −20 °C, because room temperature stored pre-gel failed gelation as no sigmoidal curve was observed (Supplementary Fig. 2). Rheological characteristics of different ECM gel concentrations and Matrigel were assessed using temperature ramping oscillatory rheology (Fig. 1h, i). All concentrations of the ECM gel and Matrigel exhibited gel-like properties when exposed to 37 °C temperature, with the storage modulus (G’) higher than the loss modulus (G”). At approximately 45 °C all gels experienced a drop in their storage modulus, indicating a melting point for the gels, including Matrigel. ECM gel at 6 mg/mL exhibited a similar rheological profile to the Matrigel, in terms of storage and loss modulus, and, for these reasons, was preferentially used for cell culture purposes. Consistently, ECM gels at 6 mg/mL concentration showed a comparable elastic modulus to Matrigel, measured by nanoindentation (Fig. 1j).

### Intestinal ECM gel proteomic profiling

**Fig. 2:**
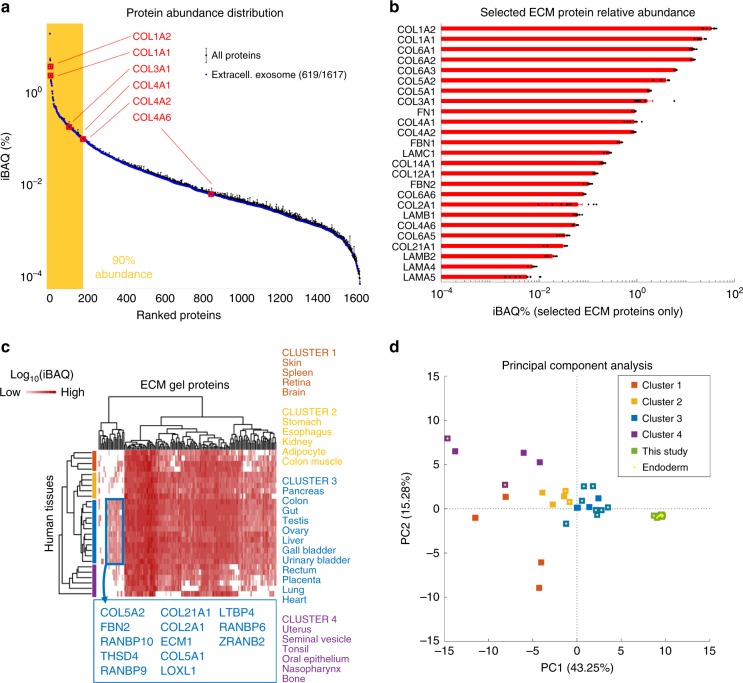
ECM proteomic analysis. **a** Protein abundance range, with 619 (on 1617 total) proteins mapped to GO-CC:0070062~extracellular exosomes highlighted. Yellow-shaded area represents the range covering 90% of total protein abundance. Collagens analyzed in Fig. 1e are also highlighted. Mean ± SEM (*n* = 3 batches, with three technical replicates each). **b** Relative abundance of selected ECM proteins. Black dots represent individual data points. Mean ± SEM (*n* = 3 batches, with three technical replicates each). **c** Hierarchical clustering analysis of mass spectrometry native human tissue data from a draft map of the human proteome, conducted for proteins in our data mapped to GO-CC:0031012~ECM. Four main clusters are identified whose color-coded tissues are reported on the right. A small group of proteins especially expressed in cluster 3 is highlighted. A fully detailed version of this heat map is reported in Supplementary Fig. 3. **d** Principal component analysis (PCA) of data from native human tissue reported in (**c**), and of data generated in this study. Tissues and samples having endodermal origin are also highlighted.

To characterize the ECM composition in terms of residual proteomic content after decellularization, a mass spectrometry analysis was performed on the powder form, before pepsin digestion^11^. More than 1600 proteins were identified, of which ~130 were recognized as derived from ECM and 619 from extracellular exosomes (Fig. 2a), confirming the dual role of ECM as a supportive structure and as a storage of adsorbed soluble signals. Exosomal proteins are most significantly over-represented in pathways related to translation and cell adhesion (Supplementary Data 1). The abundance of RNA-binding proteins within exosomes is well known and their preservation could play a role in intercellular signaling during differentiation^23^. A separate set of metabolomics experiments were performed to further analyze the residual compounds found in the pre-gel, after pepsin treatment. Expected compounds such as fatty acids residuals and sub-products of the protein metabolism were found in all three different batches analyzed (Supplementary Fig. 3a).

Despite the high diversity of identified proteins, most of them contributed for less than 1% of the total amount. Multiple collagen types were among the most abundant, mostly fibrillar (type 1, 2, 3, 5, 6), but also fibril-associated (type 12, 14, 21) and sheet forming (type 4) (Fig. 2b, Supplementary Fig. 3b).

To verify if the retained complex protein composition of the ECM gels showed similarities with specific human tissues, the ECM proteins quantified in our data were searched on a publicly available map of the human proteome (Fig. 2c). This analysis was restricted to more relevant tissues for regenerative medicine applications. The protein set given by the ECM proteins in our data (~130 proteins) was sufficient to identify a cluster of tissues that show a similar proteomic profile and comprise multiple endoderm-derived tissues, including gut, liver and pancreas (cluster 3 in Fig. 2c). A group of proteins that are almost exclusively expressed within this cluster (and in our samples) was identified. It includes not only structural constituents of the ECM, such as collagens, but also proteins responsible for cross-linking collagen fibrils and forming elastic fibers (LOXL1, FBN2). A full resolution panel of results of the hierarchical clustering analysis is reported in Supplementary Fig. 3c.

The similarity between the ECM protein composition of our decellularized matrices and the above tissues was also quantitatively investigated by principal component analysis (PCA), which showed a higher similarity of the ECM gels composition with tissues of endodermal origin (Fig. 2d). The decellularization process was able to preserve protein composition features that are not only characterizing the native tissue of the ECM gels, but also shared within a group of similar developmental origin tissues.

To assess the safety of the ECM gel, we analyzed the residual presence of galactose-alpha-1,3-galactose (alpha-gal). We screened for the presence of the antigen with immunofluorescence analyses in the non-decellularized SI mucosa, the intact DT, and six different batches of the piglet SI-derived ECM gel. We were able to assess the presence of the porcine antigen both in the fresh and in the whole DT, as already reported^24^. On the other hand, we did not observe the presence of the antigen in any of the six analyzed gels. These results showed the absence of alpha-gal in the final ECM gel, or the extreme dilution of the epitope that could not be detected (Supplementary Fig. 4).

### ECM gels support both mouse and human organoid cultures

**Fig. 3:**
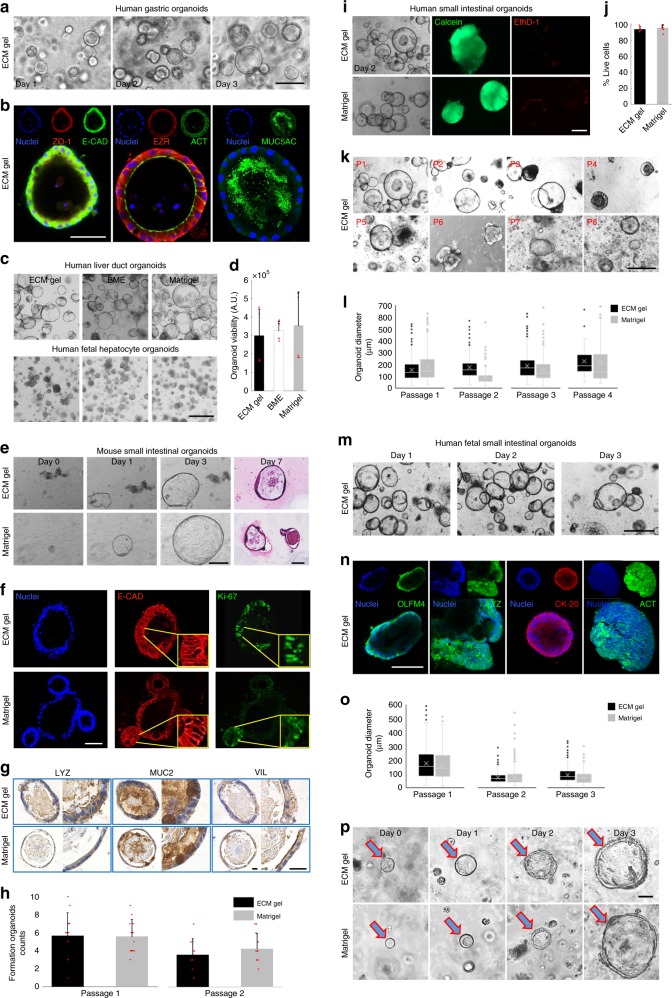
3D culture of endodermal organoids in ECM gel and Matrigel. **a** Human pediatric gastric enteroids in ECM gel. Scale bar 200 µm. **b** Planes of whole-mount immunofluorescence of 7-days gastric organoids showing both epithelial (zonula occludens-1, epithelial cadherin and actin) and gastric (ezrin and mucin-5AC) markers. Scale bar 50 µm. **c** Culture of liver ductal and hepatocyte human organoids in ECM gel, BME and Matrigel. Scale bar 500 µm. **d** Hepatocyte organoid viability. Mean ± S.D. (*n* = 8 organoid cultures). **e** Bright field and H&E images of mouse intestinal organoids in ECM gel and Matrigel. Scale bars 100 µm. **f** Immunofluorescence analysis of sections of mouse SI organoids in ECM gel and Matrigel, showing epithelial cadherin staining and proliferation marker Ki-67^+^. Scale bar 50 µm. **g** Immunohistochemical staining of mouse intestinal organoids in ECM gel and Matrigel. Scale bars 25 µm. **h** Forming mouse intestinal organoids per field of view at day 4 of culture in ECM gel and Matrigel over two passages. Mean ± S.D. (*n* = 12 organoid cultures). **i** Live/Dead assay of human pediatric SI organoids cultured in ECM gel and Matrigel. Calcein-AM shows living cells. Ethidium homodimer-1 shows dead cells. Scale bar 200 µm. **j** Quantification of vital cells from Live/Dead assay. Mean ± S.D. (*n* = 8 organoid cultures). **k** Morphology of eight consecutive passages over a period of 2 months of human pediatric SI organoids in ECM gels. Scale bar 300 µm. **l** Analyses of four consecutive passages of human pediatric SI organoids diameters at day 3 of culture in ECM gel and Matrigel. Mean ± S.D. (*n* ≈ 200 organoids). **m** Bright field of human fetal SI organoids in ECM gel. Scale bar 200 µm. **n** Whole-mount immunofluorescence Z-Planes of human fetal SI organoids showing crypt stem cell marker olfactomedin-4, crypt Paneth cell marker lysozyme, villi enterocyte marker keratin-20 and actin staining. Scale bar 100 µm. **o** Analyses of three consecutive passages of human fetal SI organoids diameters at day 3 of culture. (*n* ≈ 380 organoids). **p** Single-cell colony (arrows) formation capacity assessed over 3 days in disaggregated human fetal SI organoids in ECM gel and Matrigel. Scale bar 25 µm. Box plots are represented with the central line indicating the median of values, bounds of box indicating first and third quartiles, and whiskers to show minimum and maximum outside the first and third quartiles.

We then demonstrated the possibility of ECM gels to host different endoderm-derived organoids cultures. We therefore provided extensive analysis on both human and mouse organoid cultures, from different organs. First, human organoids of gastric origin showed high level of adaptation to the SI ECM gel. The pediatric stomach enteroids maintained the expression of both epithelial (zonula occludens-1, epithelial cadherin and f-actin) and gastric (ezrin and mucin-5AC) markers after 7 days of culture (Fig. 3a, b).

We also explored different endodermal organoids such as human liver ductal and human fetal hepatocytes organoids from different donors and compared morphology with two different standard controls, i.e., BME and Matrigel (Fig. 3c)^25,26^. No significant difference was observed when culture viability was assessed in the three different conditions (Fig. 3d).

Then we showed how Lgr5^+^ intestinal stem cells, isolated from the crypts of the mouse SI, survived and maintained their phenotype, forming expanding enteroids in the ECM hydrogel over time (Fig. 3e). Proliferating epithelial cells expressing Ki-67 were present both in ECM gels and Matrigel (Fig. 3f). Moreover, cells in ECM showed comparable expression respect to the control of intestinal differentiation markers such as mucin-2 and villin, with a higher prevalence of lysozyme (marking Paneth cells) in ECM gel compared to Matrigel cultured organoids (Fig. 3g). The formation of new organoids after split showed no significant difference between ECM gel and Matrigel over the first two passages (Fig. 3h).

Importantly, similar features were observed when human pediatric SI organoids were cultured in ECM gels. Live/Dead assay showed a comparable number of living cells to those cultured in Matrigel, demonstrating cytocompatibility of ECM gels, with standard viability potential (Fig. 3i, j). We showed that ECM gel can also sustain over time human SI cultures, with morphological analyses in 8 consecutive passages, over a time span of 2 months. A decrease in organoid morphological quality was observed in the last three passages (Fig. 3k). Nonetheless, during the first four passages, organoids maintained constant dimensions between ECM gel and Matrigel (Fig. 3l).

We then explored the possibility of ECM gels to host human organoid cultures of fetal origin. SI enteroids showed differentiated morphology (Fig. 3m) and high expression of both crypt (olfactomedin-4 and lysozyme) and villi region (cytokeratin-20/actin) proteins (Fig. 3n). Also in this case, organoids maintained comparable dimensions between ECM gel and Matrigel over multiple passages (Fig. 3o). We also confirmed the possibility to split the organoids at single cells, allowing a de novo colony formation after passaging (Fig. 3p). We demonstrated the possibility to derive organoids from pediatric donors directly in ECM gel, without ever passaging them in Matrigel. We showed the formation of new organoids from human gastric and human SI biopsies (Supplementary Fig. 5).

As a future perspective, it might be important to modulate the physical and biochemical properties of the ECM gel by adding further hydrogel-forming components. By using a synthetic hydrogel system made of poly-acrylamide, we produced a highly homogeneous hydrogel in which the collagen fibers are uniformly interspersed (Supplementary Fig. 6a) and in which the fine-tuning of gel stiffness could be easily achieved (Supplementary Fig. 6b). Poly-acrylamide, normally cell-repellent, when loaded with ECM gel, allowed cell adhesion and showed the possibility to culture human and mouse SI organoids as monolayers of cells, with an epithelial morphology (Supplementary Fig. 6c–d).

### ECM gel culture human organoid transcriptomic profile

**Fig. 4:**
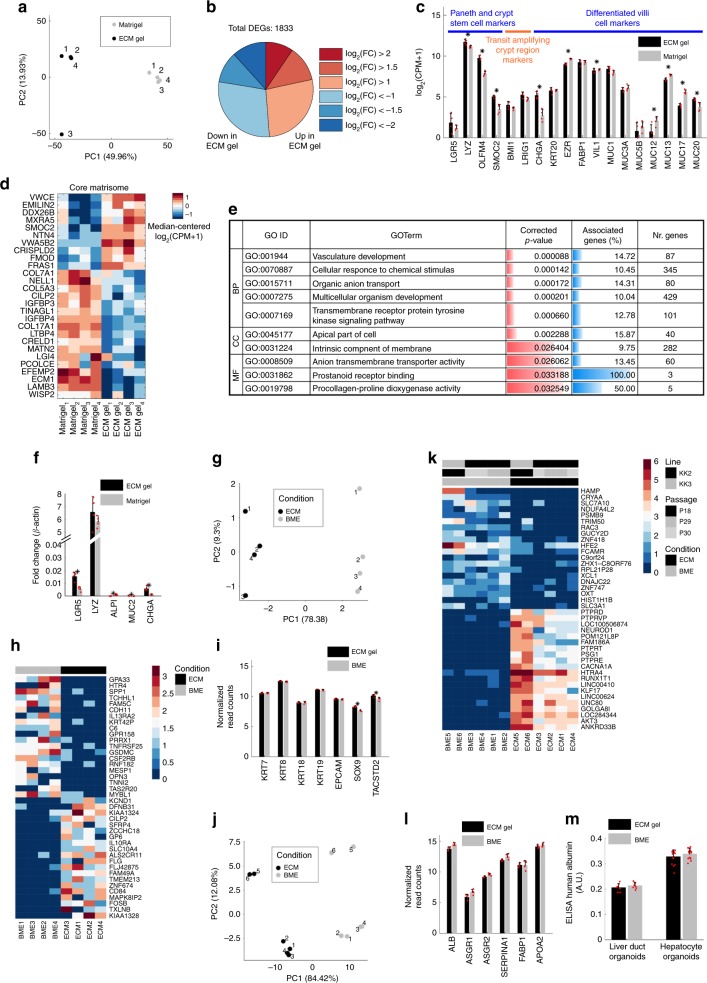
Transcriptomic analysis results of different ECM organoids. **a**–**e** Pediatric SI organoids. **a** PCA analysis. **b** Number of DEGs upregulated and downregulated in ECM compared to Matrigel for different absolute log-fold change ratios. **c** Expression of genes selected for their involvement in the indicated processes. Mean ± S.D. (*n* = 4 biological replicates). Black asterisks indicate DEGs. **d** Heat map of expression of core matrisome DEGs ordered according to hierarchical clustering. Supplementary Fig. 6a reports the corresponding analysis for matrisome-associated transcripts. **e** Selected GO categories enriched in DEGs between ECM and Matrigel involved in the interaction of cells with the extracellular space. Full results are reported in Supplementary Data 2 and Supplementary Fig. 6b. **f** Real-time PCR analysis of SI transcripts. Mean ± SEM (*n* = 4 biological replicates). Two-sided *t*-test *p*-value < 0.05. **g** PCA plot on human ductal liver organoids cultured in ECM gel vs BME. **h** Heat map of top 20 upregulated and top 20 downregulated genes ECM gel vs BME ductal organoids. **i** Ductal liver transcripts plot comparison in ECM gel vs. BME. Mean ± S.D. (*n* = 4 biological replicates). Black asterisks indicate DEGs. **j** PCA plot on human fetal hepatocyte organoids cultured in ECM gel vs. BME. **k** Heat map of top 20 upregulated and top 20 downregulated genes ECM gel vs BME hepatocyte organoids. **l** Hepatic transcripts plot comparison in ECM gel vs. BME. Mean ± S.D. (*n* = 4 biological replicates). **m** Comparison of ALB expression in ductal and hepatocyte organoids by ELISA assay. Mean ± S.D. (*n* = 3 biological, with four technical replicates each). Red dots on the bar charts represent single data points throughout the figure.

To better understand the behavior of human organoids in ECM gel compared to Matrigel, we further characterized the cultures through transcriptomic and functional analyses. We performed 3′ RNA-sequencing on human SI organoids derived from a pediatric donor (Fig. 4a–e). PCA showed that, despite sample-to-sample variability, the two groups of samples were clearly separated according to the first principal component (Fig. 4a). To better address the origin of this separation between ECM gel- and Matrigel-cultured SI organoids, we analyzed in detail the Differentially Expressed Genes (DEGs). 1833 genes were found differentially expressed, but only 388 had an absolute fold change greater than 2 (highly differentially expressed), of these 173 and 215 were upregulated and downregulated in ECM gel compared to Matrigel, respectively (Fig. 4b). To screen the importance of these DEGs in SI biological mechanisms, we selected some gene sets related to processes that could be relevant for cell adaptation and differentiation within organoids^27^. As shown in Fig. 4c, many of these genes were differentially expressed in ECM gel organoids compared to Matrigel cultured ones. Interestingly, while *LGR5* was comparable in both conditions, other crypt markers such as *OLFM4, SMOC2*, and *LYZ* were statistically overexpressed in ECM gel cultured organoids. Transit amplifying region markers (*BMI1, LRIG1*) were comparable with Matrigel controls. Differentiated intestinal cell markers were partly comparable between the two conditions (*FABP1, MUC1, MUC3A, MUC5B*) or slightly overexpressed in Matrigel compared to ECM gel cultured organoids (*EZR, VIL1, MUC12, MUC13, MUC17, MUC20*). Of the differentiation markers, only *CHGA* resulted overexpressed in ECM gel.

We connected these transcriptomic outcomes on the SI organoids with the proteomic analyses on the ECM gel to try to decipher the contribution of a more biomimetic microenvironment on the physiology of the intestinal cells. Of all preserved proteins identified in the decellularized ECM, 109 (~6% of all DEGs) were also identified as differentially expressed at the transcriptomic level, with 42 upregulated in ECM and 67 upregulated in Matrigel (Supplementary Data 2). As for the transcripts known to be in the core matrisome^28^ (Fig. 4d), the only four transcripts (*TINAGL1, LTBP4, CRELD1, ECM1*) that were both DEGs and identified as proteins, were all upregulated in organoids cultured in Matrigel. Only *TINAGL1* (IPI00115458) was identified in Matrigel in a previous proteomic study^29^. Moreover, two (*LTBP4, ECM1*) of these four transcripts were highlighted in Fig. 2c as characterizing cluster 3, the one mainly incorporating endoderm-derived tissues. Only five transcripts (*LGALS1, LGALS3, LMAN1, P4HA1, TGM2*), out of the 109, belong to the matrisome-associated gene set, and were also all upregulated in Matrigel organoids at the transcriptomic level, despite four of these proteins have been previously identified also in Matrigel (Lgals1, IPI00229517; Lgals3, IPI00131259; Lman1, IPI00132475; P4ha1, IPI00272381). Matrisome-associated (e.g., remodeling enzymes) differentially expressed genes complete panel is shown in Supplementary Fig. 7a. An unbiased analysis of GO categories over-represented in the identified DEGs at transcriptomic level highlighted multiple functional categories related to processes occurring at the cell-extracellular environment interface (Fig. 4e, Supplementary Fig. 7b, and Supplementary Data 2). Interestingly, some of the identified processes could be relevant in the ECM gel role of organoid support, like those involved in Vasculature development or Multicellular organism development. These outcomes on human SI organoids outline the strict connection between microenvironment and cell physiology, therefore moving to a native ECM might benefit organoid phenotype. We also checked by real-time PCR the differential expression of some major intestinal markers observed in the SI RNA-sequencing in Fig. 4c (Fig. 4f). In this analysis, *LGR5* and *CHGA* resulted overexpressed in ECM gel, while *LYZ* was comparable. *ALPI* and *MUC2* were both overexpressed in Matrigel. These data confirm the previous observation of a higher fraction of crypt/stem cells present in ECM-cultured human SI organoids.

Moreover, we report a full set of transcriptomic data on human liver cells. For this, we analyzed human adult liver ducts, and human fetal hepatocyte organoids, previously presented in Fig. 3c. We performed bulk 3′ RNA-sequencing with comparison of the 2 liver cell types cultured in ECM vs BME. Regarding the RNA-seq analysis for the human ductal organoids, while the PCA plot and the heatmap of the differentially expressed genes (Fig. 4g, h) showed that the organoids cultured in ECM gel were slightly different from those cultured in BME (based on PC1), none of the critical ductal markers (*KRT7, KRT8, KRT18, KRT19, EPCAM*) were downregulated (Fig. 4i). We observed how *SOX9* and *TACSTD2 (TROP2)* were significantly upregulated in the ECM gel culture condition (Fig. 4i). Both are markers of progenitor-like cells, where *TROP2* has been recently described as a marker of bipotent progenitors^30^. The cluster map of human ductal liver organoids cultured in ECM gel vs. BME is shown in Supplementary Fig 7c.

The RNA-seq analysis for the human fetal hepatocyte organoids highlighted also in this case a distance between ECM gel and BME cultured organoids, as shown in the PCA plot and in the heatmap of the differentially expressed genes (Fig. 4j, k). In this analysis we compared two separate fetal lines, KK2 and KK3, and the observed distance might also be ascribed to donor-related differences. Nonetheless, none of the specific hepatocyte markers^26^ (*ALB, ASGR1, ASGR2, SERPINA1, FABP1, APOA2*) showed any differential expression (Fig. 4l). We reported the cluster map of human fetal hepatocyte organoids cultured in ECM gel vs BME in Supplementary Fig. 7d.

Interestingly, the functional analysis on the production of human albumin by both ductal^25^ and hepatocyte^26^ organoids showed a comparable secretion in both ECM gel and BME cultures (Fig. 4m).

### In vivo delivery of ECM cultured organoids

**Fig. 5:**
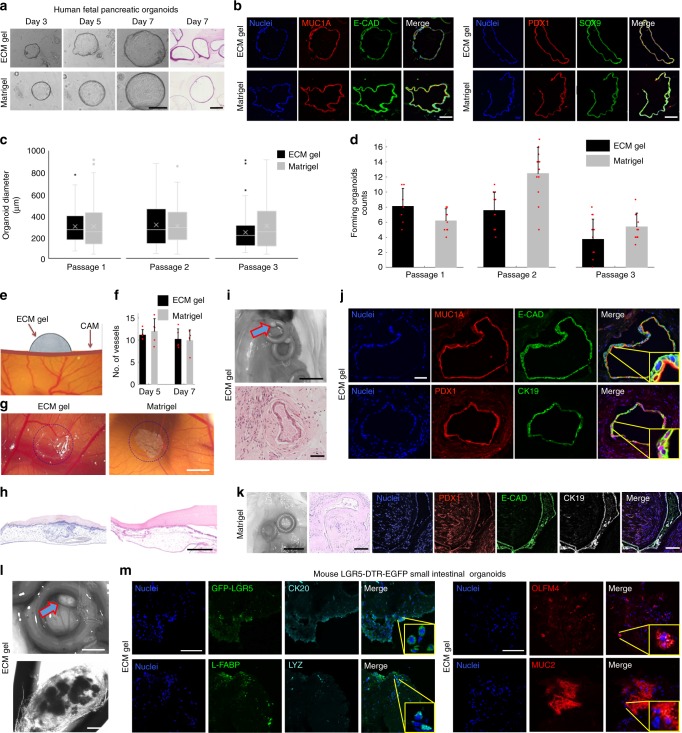
In vivo delivery of ECM gel-cultured organoids. **a** 3D culture of human fetal pancreatic ducts in ECM gel and Matrigel. Bright field and H&E images of the pancreatic organoids. Scale bars 100 µm. **b** Immunofluorescence analysis of sections of pancreatic organoids in ECM gel and Matrigel, showing expression of mucin-1A, epithelial cadherin, together with insulin promoter factor 1 and cytokeratin-19. Scale bar 50 µm. **c** Analyses of three consecutive passages of pancreatic organoid diameters at day 6 of culture in ECM gel and Matrigel. Mean ± S.D. (*n* ≈ 55 organoids). Box plots are represented with the central line indicating the median of values, bounds of box indicating first and third quartiles, and whiskers to show minimum and maximum outside the first and third quartiles. **d** Forming pancreatic ducts per field of view at day 6 of culture in ECM gel and Matrigel. Mean ± S.D. (*n* ≈ 10 organoid droplets). **e** Evaluation of the ECM gel angiogenic potential through Chick Chorioallantoic Membrane (CAM) Assay. **f** Quantification of the number of blood vessels directed towards the gel on the CAM in ECM gel and Matrigel. Mean ± S.D. (*n* = 5 biological replicates). **g** ECM gel and Matrigel on the CAM, circled in blue. Scale bar 1 mm. **h** H&E staining of the CAM in ECM gel and Matrigel. Scale bar 250 µm. **i** Mouse subcutaneous transplantation of human fetal pancreatic ducts in ECM gels. (Top) Recovery of silicon rings from mouse back with ECM gels (arrow) after 2.5 weeks. Scale bar 5 mm. (Bottom) H&E staining of pancreatic ducts after in vivo transplantation in ECM gel. Scale bar 100 µm. **j** Immunofluorescence staining of pancreatic organoids in ECM gel after 2.5 weeks in vivo, expression of pancreatic markers mucin-1A, epithelial cadherin, insulin promoter factor 1 and cytokeratin-19. Scale bar 25 µm. **k** Recovery of silicon rings with pancreatic ducts in Matrigel after 2.5 weeks (left scale bar 5 mm), H&E staining, and immunofluorescence staining (central/right scale bars 100 µm). **l** Mouse subcutaneous transplantation of murine LGR5-DTR-EGFP SI organoids in ECM gels. (Top) Recovery of silicon rings from mouse back with ECM gels (arrow) after 4 weeks. Scale bar 1 mm. (Bottom) Bright field image of ECM gel with intestinal organoids inside. Scale bar 200 µm. **m** Immunofluorescence staining of SI organoids in ECM gel after 4 weeks in vivo, showing crypt/stem markers anti-GFP-LGR5, olfactomedin-4 and lysozyme, together with villi/differentiation markers cytokeratin-20, L-type fatty acid binding protein and mucin-2. Scale bars 100 µm.

Lastly, we explored the in vivo delivery of cultured human organoids, which remains challenging as it is linked to efficient vascular support, and the possibility of using clinically compatible vectors. For this purpose, we used human fetal pancreatic organoids. Fetal ducts grown in vitro in ECM gel and Matrigel showed similar morphology (Fig. 5a). ECM gel allowed maintenance of similar expression of lineage-specific markers such as mucin-1a, epithelial cadherin, together with pancreatic-duodenal homeobox 1 (PDX1) and SOX9 (Fig. 5b). Cells were successfully expanded for at least three passages during which they maintained comparable organoid size (Fig. 5c) and number in ECM gel and control (Fig. 5d).

In order to evaluate the angiogenic potential of ECM gels, the Chick Chorioallantoic Membrane (CAM) assay was performed (Fig. 5e). When compared to Matrigel over 5 and 7 days, ECM gel showed no difference in the number of new vessels formed (Fig. 5f). Morphological (Fig. 5g) and histological (Fig. 5h) characterization of the ECM gel and control on the CAM also showed no difference.

To further evaluate the in vivo potential, we also seeded pancreatic organoids within ECM gels and Matrigel as control, and transplanted them subcutaneously in immunodeficient mice. Cells were then harvested at 2.5 (Fig. 5i) and 8 weeks (Supplementary Fig. 8a–b). At both time points, we observed the preservation of organoid organization and comparable expression of epithelial (e-cadherin), pancreatic (mucin-1A with polarized luminal localization, and cytokeratin-19) markers, and transcription factors (insulin promoter factor 1) between ECM gel and Matrigel (Fig. 5j, k, Supplementary Fig. 8c).

As an additional application of this model, we performed a set of in vivo experiments with SI organoids. To this end, we used the *LGR5-DTR-EGFP* mouse model^31^. We derived mouse SI organoids with GFP-reporter crypt stem cells which could be traced after an in vivo transplant. We transplanted these cells in ECM gels, into mice back sub-cutaneous pockets. After one month, we were able to retrieve all 5 ECM gels transplanted, which contained matured organoids (Fig. 5l). Retrieved cells showed an active stem compartment highlighted by the presence of anti-GFP for LGR5^+^ cells, double checked with olfactomedin-4. Paneth cells were present (marked with lysozyme), and we highlighted also the presence of differentiated cell types such as enterocytes and goblet cells, marked with L-type fatty acid binding protein (L-FABP), cytokeratin-20 and mucin-2 (Fig. 5m).

## Discussion

While research in the organoid field is leading to exciting findings with broad therapeutic potential, their clinical translation is greatly limited by the lack of GMP-compatible conditions for organoid derivation and expansion. We describe here the successful development of ECM gels that have the potential to both direct and influence human organoids behavior in vitro and in vivo. This includes directing cell adhesion, survival, proliferation, and differentiation, while also providing a mechanical support to the cells. An ex vivo 3D cell culture support should ideally recapitulate aspects of this native microenvironment and facilitate these functions^32^. LGR5^+^ cells, isolated from the crypts of the intestine are an example of a cell type that favors a 3D environment for ex vivo culture over 2D^33^. A 2D culture, provides an unnatural environment for the cells. In a monolayer culture, only a portion of the cell surface is in contact with ECM and neighboring cells, with the remaining portion exposed to the culture media. This provides a homogeneous supply of nutrients, cytokines and growth factors to this external membrane, which unlikely resemble the dynamic spatial gradient of nutrient supply received in vivo^34^.

Both natural and synthetic hydrogels have been examined for their ability to support organoid culture, each having its own associated advantages and limitations. Recently, synthetic alternatives to Matrigel have been reported^9,10^. While synthetic gels have the advantage of being GMP-compliant and reproducible, they are limited by a lack of biological signals provided to the cells. ECM is far more complex and here we show, that this information allowed clustering within the germ layer of derivation. We have demonstrated that intestinal ECM gels can support the culture not only of intestinal organoids, but also of cells derived from other endoderm-derived tissue such as liver, stomach and pancreas.

Porcine intestine tissue was decellularized using the DET protocol, previously established on rat intestine by our group^21^. Mesentery and the external muscle layer were removed in situ using an in-house established protocol. One cycle of the DET protocol was required to remove nuclei from the scaffold which was confirmed with H&E staining along with a significant reduction in DNA content. Histological analysis confirmed the presence of collagen, elastin and GAGs post gelation. Collagen increase compared to tissue weight is a common feature following decellularization and loss in cytoplasmic compartment. Further characterization highlighted maintenance of the main collagen isoforms^8^. Spectrophotometry and rheology experiments confirmed gelation of the ECM hydrogel at all concentrations. Gelation was fastest in the 6 mg/mL concentration, which also had the rheology profile most similar to Matrigel. Gelation also preserved appropriate stiffness which is fundamental for enteroid formation, survival and differentiation.

Past works describe how ECM is not only a mere scaffold, but it is an integral determinant of tissue specificity itself. Epithelial and mesenchymal components interact during development to direct tissue morphogenesis and differentiation. The tissue development is not a cell autonomous process, but it is instead instructed by the surrounding environment^35^. Extensive proteomic analysis on the DT powder further confirmed that the major ECM components were preserved, such as the main collagen isoforms, but also non-ECM components, which may play a role in the signal transduction of the organoids cultured in the gel. SI ECM proteomic profile clusters with the main endoderm-derived organ’s ECM profiles. This characteristic suggests that this gel could also be used to support the culture of both mouse and most relevantly human organoids derived from stomach, adult (ducts) and fetal (hepatocytes) liver, adult and fetal SI mucosa, and fetal pancreas. Many exosomal proteins were also preserved within the decellularized matrix, including proteins related to cell adhesion. However, we found a large overlap between ECM proteins present within exosomes and as structural constituents of ECM, therefore we could not discriminate between these two contributions. The metabolomics analysis on the digested powder allowed us to identify sub-products of protein degradation and fatty acid residual which were expected to be found after cell membrane breakdown during decellularization process, and were confirmed in this study.

Transcriptomic analyses performed on ECM gel cultures showed how human organoids maintain their identities compared to Matrigel cultures, and this is confirmed in human SI, liver duct, and hepatocyte organoids. Interestingly, pediatric SI organoids maintained a higher proportion of crypt stem cells in SI ECM hydrogels compared to slightly more differentiated cells in Matrigel. Nonetheless, all the specific intestinal markers, defining both crypt and villi signature, were expressed in ECM gel compared to Matrigel cultured organoids, underlying the suitability of the ECM to host human cultures. On the other hand, ECM specific markers that were detected at the proteomic level in the decellularized ECM were found to be all overexpressed in Matrigel cultured SI organoids. Only few of these were previously reported to be present in Matrigel^29^. Therefore, we could speculate on this peculiar outcome, which might be ascribed to the necessity of SI organoids to produce their intestinal ECM, compared to ECM gel cultured organoids that are already integrating signals from the native SI ECM.

A translational application of ECM-derived hydrogels is currently hampered by the high variability of the lab-derived products. In our study, batch to batch variation is observed because of the biological origin of the gel. To better standardize the process we always used similar age and similar weight (3 kg) piglets of the same pure ‘Pietrain’ breed. Each new batch was then tested for ECM digestion quality, gelation quality, stability in culture medium in incubator and cytocompatibility with organoids. To further overcome this real issue, we looked at the potential of combining the ECM hydrogel with synthetic molecules. As a proof of concept we developed a combination of the intestinal ECM hydrogel with photo-polymerizable polyacrylamide to obtain a combined gel suitable for monolayer growth of mouse and human SI organoids. However, any other synthetic material can be used. For instance, Gjorevski et al.^9^ showed that enrichment of their synthetic PEG gel with major ECM components such as fibronectin enhanced mouse intestinal stem cell survival and proliferation. The same approach is likely to benefit this ECM gel in future development studies, particularly the addition of laminin, a key component of the basement membrane in the intestine^36^.

Long-term expansion requires stable and consistent cultures. During the experiments we observed a slightly reduced organoid growth after several passages (as shown in Fig. 3k), likely due to the accumulation of stiff ECM leftovers from previous passages. However, we observed comparable outcome during the first 3–4 passages, which would be sufficient for ex vivo cell expansion. Moreover, this did not affect in vivo delivery of the cells, which is ultimately one of the main objectives of the ECM gel.

In view of GMP-grade production for clinical use, it is important to underline that all the chemicals and reagents utilized during each step of the gel production pipeline are already commercially available at GMP-grade. This list is presented in the Supplementary Table 2. Importantly, as a further step towards the applicability of our gel to a clinically translatable protocol, we demonstrated the possibility to derive organoids from human biopsies without the use of Matrigel at the first passage after tissue dissociation.

Lastly, in this work we have shown as a proof of concept that human organoids can survive in vivo maintaining both structure and signature expression at protein level. Importantly, angiogenesis occurred already at 2.5 weeks post-transplantation in vivo and increased over time with no major differences between ECM gel and Matrigel. The in vivo results we presented highlight the possibility of our SI-derived ECM gel to efficiently deliver cells of both human and animal origin. Moreover, ECM gel facilitated cell survival of up to 2 months for enteroids derived from different organs, as shown with the fetal pancreatic ductal organoids, and the SI organoids. Nonetheless, to further move toward the production of a stable GMP-grade product, functional tissue integration of transplanted human organoids will need to be demonstrated. Taken together, these results have relevant consequences for future clinical applications of in vitro expanded organoids of human origin.

## Methods

### Porcine intestinal tissue collection

Porcine (Sus scrofa domesticus) SI mucosal/submucosal layers from the ‘Pietrain’ breed were used. Piglets up to 3 kg in weight were euthanized via blunt trauma once the criteria outlined by the JSR veterinary advisors had been met. Once sacrificed, the animals were transported to the lab via courier and the intestine was harvested immediately on arrival (within 6 h of euthanasia). The whole SI was harvested (duodenum, jejunum, and ileum) and the internal tube was pulled out leaving behind the external layer and mesentery. The retrieved mucosal/submucosal tissue was then extensively cleaned with pressurized water, opened longitudinally, cut into 5 cm pieces and placed in Milli-Q® (Merck Millipore) water overnight at 4 °C, on a laboratory rotator, to begin the first step of decellularization. Each batch of ECM was composed of three pooled piglets SI.

### Decellularization of porcine intestine tissue

The DET for decellularization, previously established on rat small bowel, was optimized for the porcine intestine^21^. After the first overnight wash, the tissue was decellularized with 4% sodium deoxycholate (Sigma Aldrich) for 4 h at room temperature (RT). This was followed by a washing step in Milli-Q water for 24 h at RT, with multiple water changes throughout, and then a step of 2000kU DNase-I (Sigma Aldrich) in 1 M NaCl (Sigma Aldrich) for 3 h at RT. The tissue was then placed in Milli-Q water and washed for 2 days, with multiple water changes. Wash steps are fundamental to remove any cytotoxic residual of sodium deoxycholate. A laboratory rotator was used throughout the decellularization process.

### Gelation protocol and cell inclusion

The decellularized porcine intestine was freeze dried for 72 h (Labconco FreeZone Triad Freeze Dry Systems), milled into a thin powder using a mini-mill (Thomas Wiley, mesh 40), sterilized by gamma irradiation (17 kGy for 10 h) and stored at −20 °C until further use. For gelation, the ECM powder was digested at 4, 6, 8, 10 mg/ml in pepsin/HCl solution (1 mg/ml in 0.1 M HCl) at RT for 72 h, in constant rotation. Pre-gel was then centrifuged (200–400 g for 5 min) to precipitate and discard eventual undigested particles. Acidic pre-gel solution was commonly used freshly prepared, but it could be stored at 4 °C up to 1 month, or frozen in aliquots at −20 °C for prolonged storage. For cell seeding, while working on ice and immediately prior to use, pre-gel solution was equilibrated to cytocompatible salinity adding 10% 10X PBS for mechanical tests, or 10× DMEM F/12 (Thermo Fisher) for cell culture and neutralized to physiological pH of 7.5 by addition of NaOH 10 M and thoroughly mixing, with modification of published protocols^19,20^. During these steps, cut-end pipette tips are used, to facilitate dense gel pipetting. Gel was mixed with cell pellets and aliquot in 30–40 µL droplets in Petri dish. Gelation took place in 30 min in the incubator. Organoids were cultured in 4–6 mg/mL ECM gels. Every batch of intestinal ECM powder had to be carefully tested for biological variability. Digestion potential, deoxycholate residual from bad decellularization washes, gel formation capacity, and suitability to host an organoid culture had to be tested for every new batch before proceeding to experiments.

### Tissue histology

Tissue samples were taken at random immediately post-harvesting and after each cycle of decellularization. For paraffin embedded sections, samples were fixed in 4% paraformaldehyde solution in PBS for 24 h at RT, washed in dH_2_O, dehydrated in graded alcohol, embedded and cut into 5 μm sections. For frozen sections, samples were snap frozen in liquid nitrogen, placed in OCT and cut into 7 μm sections. For ECM gel and Matrigel® Basement Membrane Matrix Growth Factor Reduced (GFR) (Corning 354230), droplets were fixed in glutaraldehyde 2% for 2 h. After fixing, another PBS wash was followed with 100–150 μL of 2% agarose solution until the droplet was fully covered. The agarose was removed, taking with it the gel droplet and stored in 70% ethanol. The agarose/hydrogel samples were then dehydrated with a series of ethanol washes with increasing concentrations followed by two xylene washes. Samples were embedded in paraffin and cut into 7 μm sections. Tissue slides were stained according to manufacturers’ instructions with Hematoxylin and Eosin (H&E) (Thermo Fisher) and Hoechst 33342 (Thermo Fisher) to determine the presence of nuclei and Picrosirius Red (PR), Elastic Van Gieson (EVG) and Alcian Blue (AB) (Thermo Fisher) to assess retention of collagen, elastin and glycosaminoglycans respectively.

### DNA and ECM quantification

Tissue samples were taken at random immediately post-harvesting and after decellularization protocol for DNA and ECM components quantification. DNA was quantified using a PureLink Genomic DNA Mini Kit (Thermo Fisher). The final concentration of DNA in the samples was measured using a NanoDrop (model NanoDrop 1000 Spectrophotometer by Thermo Fisher). ECM components were quantified using a QuickZyme Collagen assay kit (QuickZyme Biosciences) to measure the collagen, a Blyscan Sulfated Glycosaminoglycan Assay kit (Biocolor) for the glycosaminoglycans (GAGs) and a Fastin Elastin Assay kit (Biocolor) for elastin, according to manufacturers’ instructions.

### Turbidity

The turbidity of the hydrogels was assessed using spectrophotometry (*n* ≥ 5). Two hundred microliters of the hydrogel were pipetted into a 96-well plate and absorbance at 450 nm was measured at 37 °C once per min for 1 h^37^. Readings were normalized to a PBS control and then normalized using the calculation below, where NA is the final normalized absorbance, R is the absorbance reading obtained at a given time, *R*_min_ is the smallest absorbance value recorded and R_max_ is the greatest absorbance value. From the temporal data, the half-gelation time (*t* 1/2), the gelation rate (S) and the lag time (*t* lag) were derived by logistic regression.

$${\mathrm{NA}} = \frac{{R - R_{\mathrm{min}}}}{{R_{\mathrm{max}} - R_{\mathrm{min}}}}$$

### Oscillatory rheology

The neutralized gel (3 mL) was placed in between the two plates of the rheometer heated to 37 °C with a gap size of 1 mm, and a sinusoidal stress of constant maximum amplitude of 0.5 Pa applied at a frequency of 1 Hz. The resulting strain was measured for ~1 h and 30 min^37^.

Oscillatory rheology was performed as a temperature ramping study using a Discovery HR-2 rheometer (TA instruments). 1 mL of the neutralized digested hydrogel was poured onto the preheated steel peltier plate, 4 °C for Matrigel (100% concentration) and for the ECM gel. The 40 mm parallel plate is lowered to a gap of 650–800 µm, or until the gel perfectly fills the gap. The sinusoidal stress of constant 21 maximum amplitude of 50 Pa was applied at frequency of 25 Hz, with a temperature ramp from 22–37 °C for 7.5 min, a constant temperature of 37 °C for 45–75 min and finally another temperature ramp from 37–50 °C for a period of 7.5 min. G’ and G” were measured for the entire period.

### Scanning electron microscopy (SEM)

SEM-images of the cross-section, top and bottom surfaces of the ECM gel were taken to examine surface-topography of the material. All samples were fixed in 2.5% glutaraldehyde (Sigma Aldrich) washed in 0.1 M phosphate buffer (pH 7.4), post fixed with 1% OsO_4_ (osmium tetraoxide)/1.5% Potassium Ferrocyanide K_4_[Fe(CN)_6_] in 0.1 M phosphate buffer followed by a dH_2_O wash. Specimens were then dehydrated in a graded ethanol-water series to 100% ethanol (50, 60, 70, 80, 90, 95, and 100%) and critical point-dried using CO_2_ (ref. ^38^). The samples were mounted onto aluminum stubs using sticky carbon tabs, oriented so the surfaces of interest were presented to the beam. Samples were coated with a 2 nm-thin layer of Au/Pd using a Gatan ion-beam coater, and viewed using a Jeol 7401 FEG-SEM.

### CAM assay

Fertilized chicken eggs of ‘White Leghorn’ breed (Henry Stewart and Co.) were incubated in a MultiQuip Incubator (E2) at 37 °C with 60% constant humidity^39^. Ethics approval was obtained by the University College London Animal Ethics Committee. A small window was made in the shell on day 3 of chick embryo development under aseptic conditions. The window was resealed with adhesive tape and eggs were returned to the incubator until day 8 of chick embryo development. On day 8, ECM gels and Matrigel grafts were placed on top of the CAM and eggs were resealed and returned to the incubator. On day 10 PBS was added to the CAM to avoid the CAM drying out. Pictures were taken on day 13 and day 15. On day 15 ECM gel and Matrigel grafts with surrounding CAM were harvested from each embryo and fixed with 4% paraformaldehyde before paraffin embedding. Serial 5 μm sections were stained with H&E. Slides were digitally scanned using the NanoZoomer (Hamamatsu Photonics K.K.).

### Proteomic sample pre-processing

The lyophilized ECM powder batch, derived from three intestines, was split into three biological replicates, each processed independently and analyzed in triplicate by LC-MS/MS. The powder was resuspended in lysis buffer and two spike-in proteins (each at 0.5 µg/100 µg of protein powder) were added: carnitine monooxygenase oxygenase subunit (cntA, D0C9N6) from Acinetobacter baumannii, and CTP synthase (CTPsyn, Q9VUL1) from Drosophila melanogaster. Protein extraction was performed by heating at 90 °C for 10 min, and centrifuging at maximum velocity for 10 min at 4 °C. ECM-derived proteins were reduced in 0.1 M dithiothreitol (DTT) at 95 °C for 5 min, dissolved in 8 M urea solution after cooling down to RT, alkylated with 55 mM iodoacetamide for 30 min at 25 °C in the dark. Alkylated proteins were purified using Microcon YM-10 filter unit (MRCPRT010, Millipore) for eight times at 14,000 × *g* for 40 min^40^ followed by trypsin (Promega) digestion for 16 h at 37 °C. pH was adjusted to 3 by addition of formic acid. Peptides were desalted by C-18 column and dried into powder and were then re-suspended in 30 µL 0.1% acetic acid for the following mass spectrometry analysis.

### Proteomic LC-MS/MS analysis

Protein identification by liquid chromatography–tandem mass spectrometry (LC-MS/MS) was performed using Thermo Fusion Mass Spectrometer with Thermo Easy-nLC1000 Liquid Chromatography. 130 min of LC-MS gradients were performed by increasing organic proportion. The first level of MS was detected by Orbitrap with parameter of Resolution at 120 K, Scan Rang at 300–1800 *m/z*, Mass Tolerance at 10 ppm. The second level of MS was isolated by Quadrupole, activated by HCD and detected by Orbitrap. The Orbitrap Resolution for the second level of MS was 30 K.

### Proteomic bioinformatics analysis

The mass spectrometry-derived data were searched against a human protein database (Uniprot Homo sapiens reference proteome, UP000005640) by MaxQuant v. 1.6.7.0 (ref. ^41^). Oxidation of methionine residues and acetyl of protein N-term were set as variable modifications. Carbamidomethyl on cysteine was set as fixed modification. Peptide-spectrum matches (PSMs) were adjusted to a 1% and then assembled further to a final protein-level false discovery rate (FDR) of 1%. Intensity-based absolute quantification (iBAQ)^42^ was normalized according to the mean quantification of the two spiked-in proteins. Proteins with <2 unique peptides identified were filtered out. The remaining 1617 proteins were used in the subsequent analysis. Mean and standard error of the mean among replicates were calculated in MATLAB R2017a based on normalized iBAQ intensity values, then recalculated in percentage. Overrepresentation analyses of all and exosomal proteins was performed in DAVID 6.8 (ref.^43)^. Proteins from Gene Ontology - Cellular Component (GO-CC) categories ECM (GO-CC:0031012) and extracellular exosomes (GO-CC:0070062) were selected. A hierarchical clustering analysis of the expression of this ECM protein set in native tissues from a recent draft map of the human proteome^44^ (online resource available at http://www.proteomicsDB.org) was obtained using the web-based tool Expression heatmap^45^, reporting protein expressions in different tissues quantified as iBAQ at the protein level. A principal component analysis (PCA) was performed in MATLAB using log_10_ iBAQ intensity values from our data and from http://www.proteomicsDB.org, both within GO-CC:0031012.

### Metabolomics

Thirty microliters of ECM powder pepsin-digested (pre-gel) were analyzed by gas chromatography–mass spectrometry (GC-MS) based metabolomics using a standard protocol^46^ on a Thermo Trace GC and DSQ II mass spectrometer. Chromatographic deconvolution, alignment and database matching was performed using MS-DIAL 3.90 (ref. ^47^).

### Culture of mouse intestinal organoids

CD1 mice and LGR5-DTR-EGFP mice^31^ were sacrificed by cervical dislocation and the intestine was harvested from the pylorus to the caecum. The obtained tissue was washed through once with ice-cold PBS, cleared of any mesenteric or fatty tissue and cut longitudinally. Following a further series of PBS washes, a cover slip was used to shave away the villi and the remaining tissue was cut into 2–3 mm pieces and washed vigorously. This was then incubated in 2 mM ethylenediaminetetraacetic acid (EDTA) in PBS for 30 min followed by vigorous shaking for 5 min in PBS. The obtained supernatant, containing the intestinal crypts, was centrifuged at 800 rpm for 5 min at 4 °C (Hettich zentrifugen Rotina 420). The pellet was washed once with basal media (Advanced DMEM/F12 media, supplemented with 1% of each GlutaMAX, HEPES and Penicillin/Streptomycin) and centrifuged at 1000 rpm. The pellet was re-suspended in Matrigel growth factor reduced and plated onto a 24-well plate. Primocin 1× (Thermo Fisher) and ROCK inhibitor 10 µm are added after isolation. For medium recipe look in Supplementary Table 3.

### Culture of pediatric intestinal and gastric human organoids

Human pediatric samples from SI and stomach were collected after informed consent, in compliance with all relevant ethical regulations for work with human participants, following the guidelines of the licenses 08ND13 and 18DS02. SI crypt stem cells and gastric crypt stem cell were isolated from pediatric biopsies following well-established dissociation protocols^48,49^. Isolated crypts at first passage (p0) were cultured in Matrigel growth factor reduced droplets, or in 4 mg/mL ECM gel. For media recipes look in Supplementary Tables 4 and 5.

### Culture of human hepatocyte and liver duct organoids

Liver organoids were cultured following the protocol previously published^25,26^. Briefly, hepatocyte organoids were split by gentle dissociation with TrypLE Express (Thermo Fisher), while ductal organoids were passaged by manual disruption. Organoids were seeded in ECM gels, Matrigel and Cultrex® 3-D Culture Matrix™ basement membrane extract (BME), both at 100% concentration, as controls. For media recipes look in Supplementary Tables 6 and 7.

### Culture of human fetal SI organoids and pancreatic ducts

SIs and pancreases were dissected from human fetal tissue fragments obtained immediately after termination of pregnancy from 10 to 20 PCW (post conception week), in compliance with the bioethics legislation in the UK. Fetal samples were sourced via the Joint MRC/Wellcome Trust Human Developmental Biology Resource under informed ethical consent with Research Tissue Bank ethical approval (08/H0712/34 + 5 and 08/H0906/21 + 5). Similar isolation protocol for the mouse SI was adopted. For the pancreases, mesenchyme surrounding the pancreas was removed and epithelial tissue was digested in dispase II (Gibco) in Hank’s balanced salt solution (HBSS; Thermo Fisher) at 37 °C for 3 min. Further dissociation was performed using collagenase P (Sigma Aldrich) with gentle pipetting. Cell clusters were rinsed once with 4 mL of advanced Dulbecco’s modified Eagle’s medium/nutrient mixture F12 with 1% Penicillin/Streptomycin (AdDMEM/F12; +1% P/S) and several times with DMEM/F12 + 1% P/S, mixed with 30 μL of Matrigel (100% concentration), and seeded in 24-well plates. For media recipes look in Supplementary Table 4 and 8.

### Passage of organoids in ECM gel and Matrigel

Cell were passaged every 6–8 days. To passage the organoids, ECM gel and Matrigel droplets are thoroughly disrupted by pipetting in the well and transferred to tubes in ice. ECM removal can be aided with 20 min treatment of the droplets with Cell Recovery Solution (Corning) at 4 °C. Cells are washed with 10 mL of cold basal DMEM F-12 +++ (F-12 + P/S + HEPES + Glutamax) and spin at 200 × *g* at 4 °C. Supernatant is discarded. If any ECM or Matrigel is left, wash is repeated. The pellet is resuspended in 1 mL of cold basal medium and organoids are manually disrupted by narrow (flamed) glass pipette pre-wet in BSA 1% in PBS, to avoid adhesion to the glass. Cells are washed, pelleted and supernatant is discarded. Almost-dry pellets of disaggregated organoids are thoroughly resuspended either in cold liquid Matrigel or in cold ECM equilibrated gel, aliquot in 30–40 µL droplets in Petri dishes, and incubated at 37 °C for 30 min to form a gel. For single cell colony formation and for monolayer cell seeding, organoids pellets are treated with TrypLE Express for 5–7 min (depending on organoid size and type) at 37 °C and accurate pipetting. Disaggregated cells are washed, pelleted, and resuspended in culture medium with ROCK inhibitor.

### In vivo implantation

Animal work was ethically approved and carried out under Home Office Project Licence PPL PDD3A088A. NODSCID-gamma (NSG) mice were anaesthetized with a 2–5% isoflurane:oxygen gas mix for induction and maintenance. Pancreas organoids were embedded in ECM gel and Matrigel drops within sterile silicon O-rings (3.35 × 1.20). Cultures were conducted for 2–4 days before grafting subcutaneously of NSG mice. For subcutaneous transplantation, buprenorphine 0.1 mg/kg was administered at the induction for analgesia. Under aseptic conditions a midline incision (0.5 cm) was performed on the back of the mice and the ECM gel and Matrigel drops within the O-rings were inserted in lateral pockets. Mice were sacrificed at 2.5 weeks, 4 weeks, and 8 weeks post-transplant and content of the rings fixed in 4% PFA for 1 h for histological analysis.

### Quantification of stem cell colonies and organoid diameters

To quantify the colony formation in ECM gels and Matrigel, *n* ≥ 10 fields of view at 5× per replicate were acquired at the Zeiss Axio Observer A1 and counted. For organoid dimension quantification, *n* ≥ 50 full grown organoids were randomly quantified in different 5× fields of view per replicate. For a better approximation, 3 diameters per organoid were measured and mean diameter was considered in the final calculation.

### Cell viability assay

Cells were passaged to ECM gel and Matrigel and seeded. Enteroids were expanded for 2 days and tested for combined gel cytocompatibility. Viability assay was performed using LIVE/DEAD™ Viability/Cytotoxicity Kit, for mammalian cells (Thermo Fisher), following supplier instructions. Briefly, organoids were washed with basal DMEM F-12 and incubated in basal medium with hoechst, calcein-AM and ethidium homodimer-1 for 45 min. Cells in ECM gel and Matrigel droplets were washed twice and analyzed. Hepatocyte organoids vitality was analyzed through Cell Titer-Glo viability assay (Promega) following manufacturer’s instructions.

### Immunofluorescence and protein quantification

ECM gel and Matrigel droplets with embedded enteroids were fixed in 2% glutaraldehyde dissolved in PBS with Ca/Mg for 1 h at RT, and then washed. For sections, droplets were dehydrated with sucrose 30% overnight, included in OCT and cut at the cryostat microtome in 7 µm sections. Whole mount staining was performed by blocking and permeabilizing the cells with PBS-Triton 0.5% with BSA 1%. Primary antibodies were incubated in blocking buffer for 24 h at 4 °C in rotation and extensively washed. Secondary antibodies were incubated overnight at 4 °C in rotation and washed. Antibody list and dilutions are reported in Supplementary Table 9. For human albumin protein quantification, human ductal organoids and human fetal hepatocyte organoids were culture for 3 days (with no medium change) and medium was collected from each well (*n* = 4 per condition). Spent media were analyzed with Human Albumin ELISA (enzyme-linked immunosorbent assay) kit, following manufacturer’s instructions.

### Image acquisition

Mouse and human organoids were imaged using a Zeiss Axio Observer A1. Stained sections were acquired on a Leica DMIL microscope and DFC420C camera, or using a Hamamatsu Photonics NanoZoomer. Immunofluorescence images of whole mount stainings and sections were acquired on a confocal microscope Zeiss LSM 710.

### Bulk 3′ RNA-sequencing

For human pediatric SI organoids, RNA was isolated from cultured organoids in ECM gel and Matrigel with 20 min treatment of the droplets with Cell Recovery Solution (Corning) at 4 °C. Cells were then washed in ice-cold PBS to remove matrix leftovers that could interfere with RNA isolation. Organoids were centrifuged at 200 g at 4 °C and surnatant discarded. Dry pellet was lysed with RLT buffer (Qiagen). RNA was isolated with RNeasy Mini Kit (Qiagen) following manufacturer’s instructions. Total RNA (100 ng) from each sample was prepared using QuantSeq 3′ mRNA-Seq Library prep kit (Lexogen GmbH) according to manufacturer’s instructions. The amplified fragmented cDNA of 300 bp in size were sequenced in single-end mode using the Nova Seq 6000 (Illumina) with a read length of 100 bp.

For human liver ductal organoids, and human fetal hepatocyte organoids, 5 ng of RNA/sample were used as input for the library preparation following the CEL-Seq2 technique^50^. Briefly, RNA of liver organoids was extracted using the Qiagen RNeasy kit following the manufacturer’s instructions and stored at −80 °C prior to processing. Total RNA was then precipitated with 2 µg GlycoBlue (Ambion) overnight at −20 °C. Pelleted RNA were dissolved in reverse transcription reaction mix (Invitrogen) with UMI barcode primers and dNTPs (Promega) and incubated at 70 °C for 2 min. First and second strand synthesis (Ambion) was performed and sequencing samples were pooled into single library prior to in vitro transcription (Ambion). Amplified RNA was used as template to generate complementary DNA (cDNA) libraries using Illumina TruSeq primers. Libraries were sequenced on an Illumina NextSeq500 at high output using 75- bp pair-end sequencing.

### Transcriptome bioinformatic analyses

For human pediatric SI organoids, Illumina novaSeq base call (BCL) files were converted into fastq files through bcl2fastq (version v2.20.0.422) following software guide. Sequence reads were trimmed using bbduk software (bbmap suite 37.31), following software guide, to remove adapter sequences, poly-A tails and low-quality end bases (regions with average quality below 6). Alignment was performed with STAR 2.6.0a^51^ on hg38 reference assembly obtained from cellRanger website (Ensembl 93), following online site guide. The expression levels of genes were determined with htseq-count 0.9.1 by using cellRanger pre-build genes annotations (Ensembl Assembly 93). All transcripts having <1 CPM in less than four samples and percentage of multimap alignment reads >20% simultaneously were filtered out. Differentially expressed genes (DEGs) were computed with edgeR^52^, using a mixed criterion based on *p*-value, after false discovery rate (FDR) correction by Benjamini-Hochberg method, lower than 0.05 and absolute log_2_(fold change) higher than 1. A Principal Component Analysis was performed by Singular Value Decomposition (SVD) on log_2_(CPM + 1) data, after centering, using MATLAB R2019a (The MathWorks). Hierarchical clustering of ECM-related gene sets^28^ was performed with Euclidean distance and complete linkage using median-centered data, and plotted as heat maps using MATLAB. DEGs over-representation analysis of Gene Ontology (GO) categories was performed using ClueGO (version 2.5.4)^53^.

For human liver ductal organoids, and human fetal hepatocyte organoids DNA library sequencing, mapping to the human reference genome and quantification of transcript abundance were performed^50^. Briefly, bulk sequencing libraries of liver organoids were analysed using the DESeq2 package^54^. Bioinformatics analysis was performed using R version 3.4.0 (R Foundation, https://www.r-project.org) and RStudio version 1.0.143 (https://www.rstudio.com).

### Real-time PCR

cDNA was prepared using High-Capacity cDNA Reverse Transcription Kit (Applied Biosystems, #4368813). Quantitative PCR detection was performed using PowerUp™ SYBR® Green Master Mix (Applied Biosystems, A25742). Assays for each sample were run in triplicate and were normalized to housekeeping gene β-actin, where data was expressed as Mean ± SEM Primer sequences are listed below:

**Table:** 

Primer name	Forward	Reverse
human β-ACTIN	TTCTACAATGAGCTGCGTGTG	GGGGTGTTGAAGGTCTCAAA
human LGR5	CAGTGCAGTGTTCACCTTCC	AGTGCCAGAACTGCTATGGT
human LYZ	CATTGTTCTGGGGCTTGTCC	TCATTACACCAGTAGCGGCT
human MUC2	CAACAACTCCGAAGCTGTG	CAAATGTTTCTCGGTCACC
human ALPI	TCATCATGAGGGTGTGGCTT	TGTAGGCTTTGCTGTCCTGA
human CHGA	GAAGAAGGCCCCACTGTAGT	AGTGCTCCTGTTCTCCCTTC

### Stiffness measurement

Stiffness measures were taken at the Piuma Nanoindenter (Optics 11) on Petri-dishes with 30 µL ECM gels and Matrigel droplets immersed in PBS. The probe parameters used for the measures were: tip radius 57 µm, and probe stiffness 0.44 N/m. For the combined hydrogels (polyacrylamide with ECM gel) an Atomic Force Microscope was used (XEBio, Park Systems, Korea). Force-displacement curves were acquired using PPP-CONTSCR-10 pyramidal tips mounted on Si_3_N_4_ cantilevers with a nominal spring constant of 0.2 N/m (NanoSensors, Neuchatel, Switzerland). Cantilever spring constants were calibrated by the manufacturer prior use. Indentations were performed at a rate of 0.5 µm/s. All AFM measurements were done in a fluid environment (PBS) at room temperature. The Young’s modulus was calculated applying a fit of the Hertz model to the force curve, assuming a Poisson ratio of 0.5.

### Co-polymerized hydrogels

Polyacrylamide pre-polymer is prepared by mixing acrylamide/bis-acrylamide, 40% solution 29:1 (Sigma Aldrich) with PBS−/− and photo-initiator irgacure 2959 (Ciba) solved at 35 mg/mL in methanol (Sigma Aldrich). For 1 mL of a 20% final acrylamide concentration, 100 µL of irgacure, 500 µL acr/bis-acr solution and 400 µL of PBS are mixed and kept in the dark until use. Neutralized 10 mg/mL ECM pre-gel is allowed to gelate in incubator for 30 min. The gel is then disaggregated by repetitive pipetting and thoroughly mixed with polyacrylamide pre-polymer with proportions 25–75, 50–50, 75–25. Liquid pre-gel is then polymerized between two cover glasses and a silicon ring by photoactivation at the DYM40183 BlueWave 75 UV curing spot lamp. Co-polymerized hydrogel is then extensively washed in PBS with Pen-Strep to remove any cytotoxic acrylamide monomer from the gel bulk. Prior to use for cell seeding, co-polymerized hydrogels are cut and positioned in culture wells, and pre-equilibrated with basal medium overnight.

### Statistical analysis

Statistical analyses were performed using the following software: MATLAB (v. R2017a) for PCA, pie plot, bar plot, hierarchical clustering with proteomic and RNA-seq data, Microsoft Excel Professional Plus (v. 2016 MSO) to normalize and filter the proteomic data, GraphPad Prism Mac (v. 6.0 h) was used with all other graphs and charts.

### Reporting summary

Further information on research design is available in the Nature Research Reporting Summary linked to this article.

## Supplementary information

Supplementary Information

Reporting Summary

Description of Additional Supplementary Files

Supplementary Data 1

Supplementary Data 2

## Data Availability

The authors declare that all data supporting the findings of this study are available within the article, its Supplementary Information, attached files, and online deposited data (Proteomic data: MassIVE MSV000084136; SI RNA-seq data: GSE135108, Liver RNA-seq data: GSE138611), or from the authors upon reasonable request.
