# Supplementary material for: Extracellular matrix hydrogel derived from decellularized tissues enables endodermal organoid culture
Source: Nat Commun. 2019 Dec 11;10:5658. doi: 10.1038/s41467-019-13605-4 (PMC6906306; doi:10.1038/s41467-019-13605-4)
Supplement: Supplementary file 1 — Supplementary Information [file 41467_2019_13605_MOESM1_ESM.pdf]

Supplementary Figures and Tables

Extracellular matrix hydrogel derived from decellularized tissues enables endodermal organoid culture

Giovanni Giuseppe Giobbe[1,¶], Claire Crowley[1,¶], Camilla Luni[2¶], Sara Campinoti[1,3], Moustafa Khedr[1], Kai Kretzschmar[4], Martina Maria De Santis[1], Elisa Zambaiti[1], Federica Michielin[1], Laween Meran[1,5], Qianjiang Hu[2], Gijs van Son[4], Luca Urbani[1], Anna Manfredi[6], Monica Giomo[7], Simon Eaton[1], Davide Cacchiarelli[6], Vivian S. W. Li[5], Hans Clevers[4,8], Paola Bonfanti[1,3], Nicola Elvassore[1,2,7,*] & Paolo De Coppi[1,9,*].

[1] Stem Cell and Regenerative Medicine Section, University College London GOS Institute of Child Health, London, UK

[2] Shanghai Institute for Advanced Immunochemical Studies (SIAIS), ShanghaiTech University, Shanghai, China

[3] Epithelial Stem Cell Biology & Regenerative Medicine Laboratory, the Francis Crick Institute, London, UK

[4] Oncode Institute, Hubrecht Institute, Royal Netherlands Academy of Arts and Sciences (KNAW) and University Medical Center (UMC) Utrecht, Utrecht, Netherlands

[5] Stem Cell and Cancer Biology Lab, the Francis Crick Institute, London, UK

[6] Telethon Institute of Genetics and Medicine (TIGEM), Pozzuoli, Italy

[7] Dept. of Industrial Engineering, University of Padova, Padova, Italy

[8] Princess Máxima Center (PMC) for Pediatric Oncology, Utrecht, Netherlands

[9] Dept. of Specialist Neonatal and Paediatric Surgery, Great Ormond Street Hospital, London, UK

¶ These authors contributed equally: Giovanni Giuseppe Giobbe, Claire Crowley, Camilla Luni

* These authors jointly supervised this work: Nicola Elvassore, n.elvassore@ucl.ac.uk, and Paolo De Coppi, p.decoppi@ucl.ac.uk.

Supplementary Figures

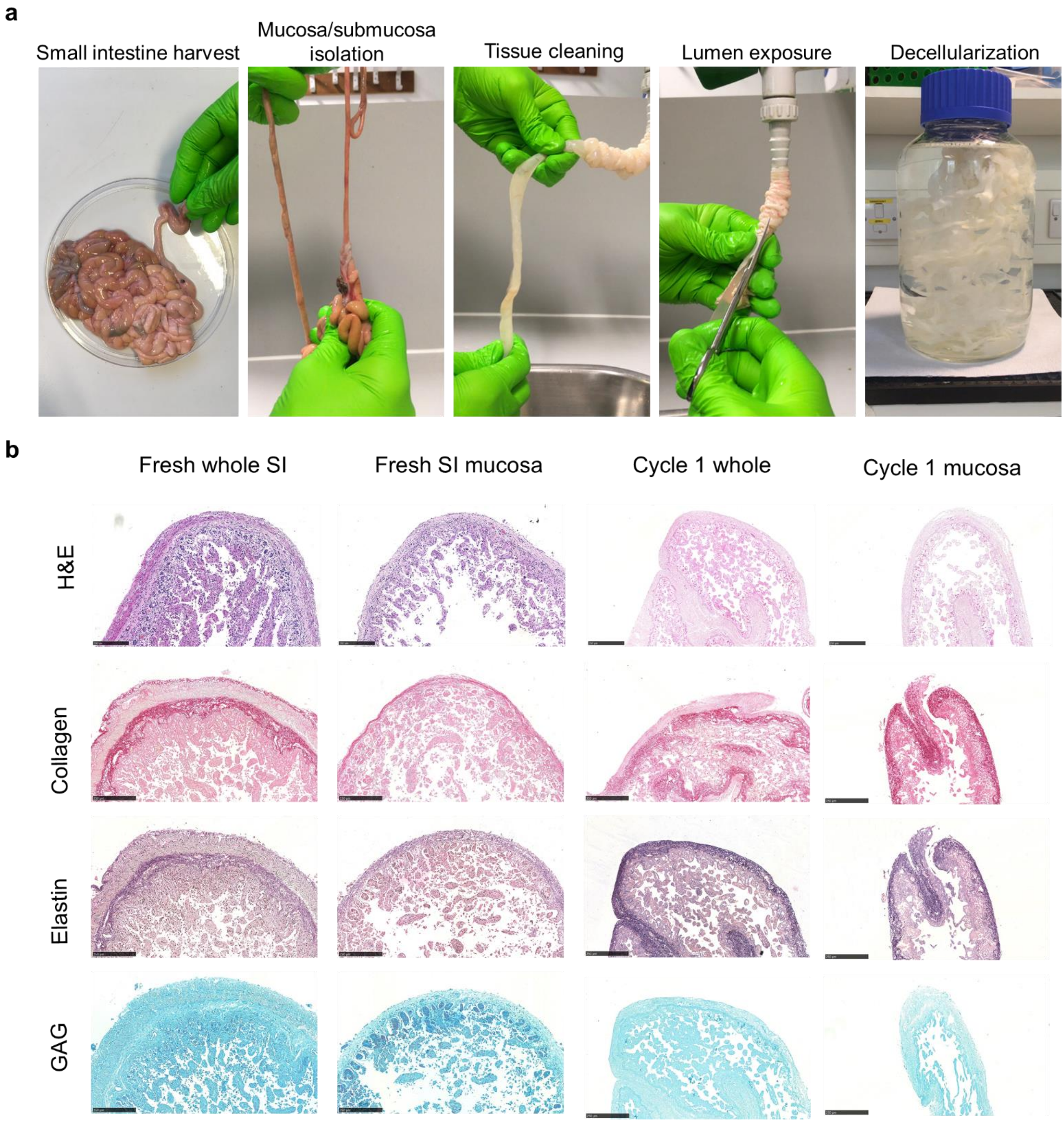

Supplementary Figure 1: (a) Images of the laboratory procedure for piglet small intestine mucosa/submucosa decellularization. (b) Sus scrofa small intestine sections pre- and post-decellularization staining. Hematoxylin/eosin, Picrosirius Red, Verhoeff's and Alcian Blue for cell nuclei, collagen, elastin and glycosaminoglycans, respectively. The images show complete removal of antigenic cellular material, and high preservation quality of extracellular matrix proteins after the process of decellularization. Scale bars 250 µm.

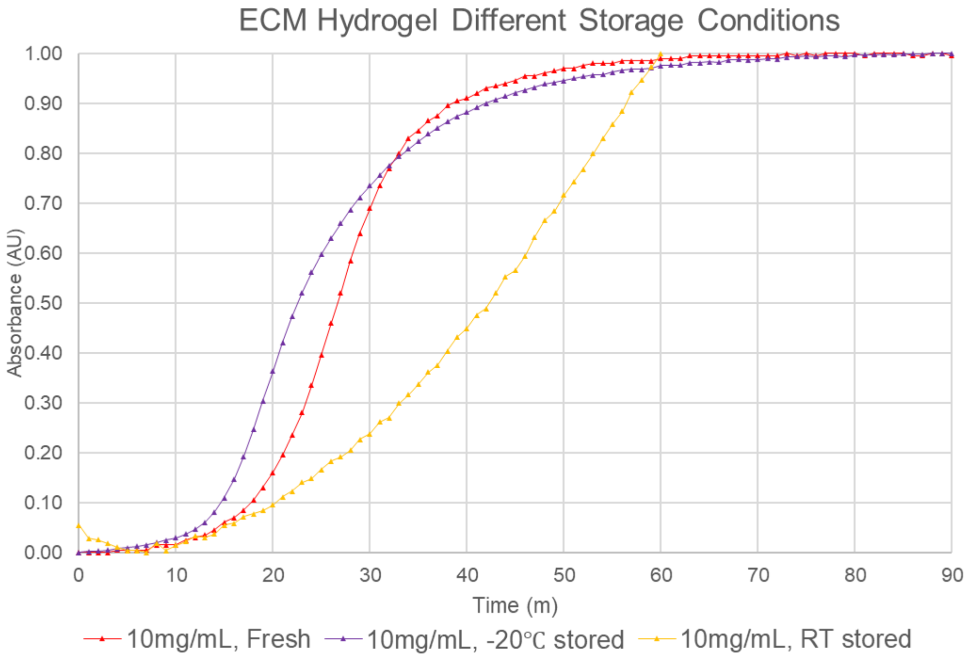

Supplementary Figure 2: Spectrophotometry graphs representing sample gelation kinetics for 10 mg/mL ECM gels freshly prepared at 4ºC, stored at -20ºC, and stored at room temperature for 1 month. No statistically significant difference in the T_{lag} is observed between the freshly prepared and the -20ºC stored. RT stored gel fails gelation as no sigmoidal curve is observed. Values are mean of 2 biological replicates, 7 technical replicates each. Student t-test p-value ≤ 0.05.

a

Quantmass	Compound Name
117	lactic acid
147	Glycolic acid
115	2-hydroxypentanoic acid
147	5,6-Dihydrouracil
220	urea
144	L-Valine
233	4-hydroxybutyric acid
171	Urea
248	beta alanine
174	Ethanolamine
233	Oxamic acid
314	leucine
286	Glycerol
299	Phosphate
158	L-Isoleucine
174	Glycine
214	putrescine minor
184	uracil
245	Fumaric acid
218	L-Threonine
174	O-Acetyl-L-serine; GC-EI-TOF; MS; 2 TMS; BP
255	Thymine
243	Glutaric acid(or anhydride)
234	2,3-Bisphospho-glyceric acid; GC-EI-TOF; MS;
304	3-aminoisobutyric acid
156	oxoproline
146	phenylalanine minor
244	5-aminovaleric acid
290	N-acetylaspartic acid
257	Lauric acid; GC-EI-TOF; MS; n TMS; RT
174	Putrescine; GC-EI-TOF; MS; 4 TMS; BP
189	Xylose_1_minor+Lyxose_1_minor
174	Ornithine_3TMS
174	N-Acetylputrescine; GC-EI-TOF; MS; 2 TMS; BP
174	Gly-Gly; GC-EI-TOF; MS; 4 TMS; BP
285	Myristic acid
179	Adenine; GC-EI-TOF; MS; 2 TMS; BP
179	tyrosine minor
204	z hexose perTMS
179	tyrosine minor
217	L-Iditol; GC-EI-TOF; MS; n TMS; BP
174	L-(+)-Lysine; GC-EI-TOF; MS; n TMS; RT
299	L-Tyrosine; GC-EI-TOF; MS; n TMS; RT
204	Pentadecanoic acid(15:0)
295	hexose perTMS NIST
117	Pyridoxine; GC-EI-TOF; MS; 3 TMS; BP
313	palmitoleic acid
202	tryptophan 3TMS
339	palmitic acid
341	oleic acid
202	STEARIC ACID; EI-B; MS
217	N-acetyl-D-tryptophan minor2
372	leucrose
246	1-monopalmitin
217	N-Acetylneuraminic acid; GC-EI-TOF; MS;
397	Sucrose; GC-EI-TOF; MS; 8 TMS; BP
361	Behenic acid; GC-EI-TOF; MS; n TMS; RT
334	beta-gentiobiose
191	b-Lactose_2_minor
81	lactobionic acid 2
204	squalene
69	CAMPESTEROL; EI-B; MS
483	Lignoceric acid; GC-EI-TOF; MS; n TMS; RT
503	LANOST-8-EN-3-OL; EI-B; MS
453	tocopherol beta NIST
329	Hexacosanoic acid
255	cholesterol
207	deoxycholic acid
203	cholic acid

b

Protein names	Gene names	Unique peptides	Sequence coverage [%]
Collagen alpha-2(I) chain	COL1A2	15	9.1
Collagen alpha-1(I) chain	COL1A1	17	11.7
Collagen alpha-1(VI) chain	COL6A1	21	22.3
Collagen alpha-2(VI) chain	COL6A2	16	15.7
Collagen alpha-3(VI) chain	COL6A3	39	13.2
Collagen alpha-2(V) chain	COL5A2	10	8.9
Collagen alpha-1(V) chain	COL5A1	7	5.7
Collagen alpha-1(III) chain	COL3A1	4	7.5
Fibronectin	FN1	39	21.3
Collagen alpha-1(IV) chain	COL4A1	5	4
Collagen alpha-2(IV) chain	COL4A2	6	4.4
Fibrillin-1	FBN1	45	21.8
Laminin subunit gamma-1	LAMC1	24	17.3
Collagen alpha-1(XIV) chain	COL14A1	17	11.4
Collagen alpha-1(XII) chain	COL12A1	26	12.1
Fibrillin-2	FBN2	24	12.9
Collagen alpha-6(VI) chain	COL6A6	8	2.9
Collagen alpha-1(II) chain	COL2A1	2	1.3
Laminin subunit beta-1	LAMB1	10	6.3
Collagen alpha-6(IV) chain	COL4A6	4	4.5
Collagen alpha-5(VI) chain	COL6A5	3	1.2
Collagen alpha-1(XXI) chain	COL21A1	3	2.9
Laminin subunit beta-2	LAMB2	7	5.8
Laminin subunit alpha-4	LAMA4	2	1.4
Laminin subunit alpha-5	LAMA5	4	1.2

c

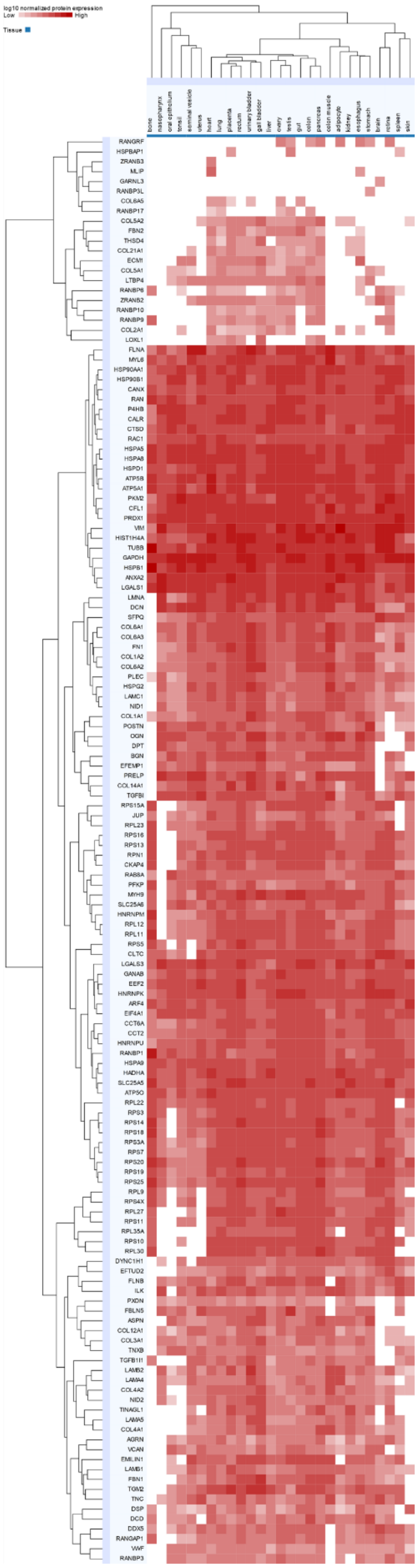

Supplementary Figure 3: (a) Metabolomics screening. List of compounds found in all 3 different batches of SI ECM pre-gel analyzed. (b) Number of unique peptides and protein sequence coverage for the ECM proteins shown in Figure 2B. (c) Full resolution results of the hierarchical clustering analysis reported in Figure 2C, image has been 90-degree rotated for clarity.

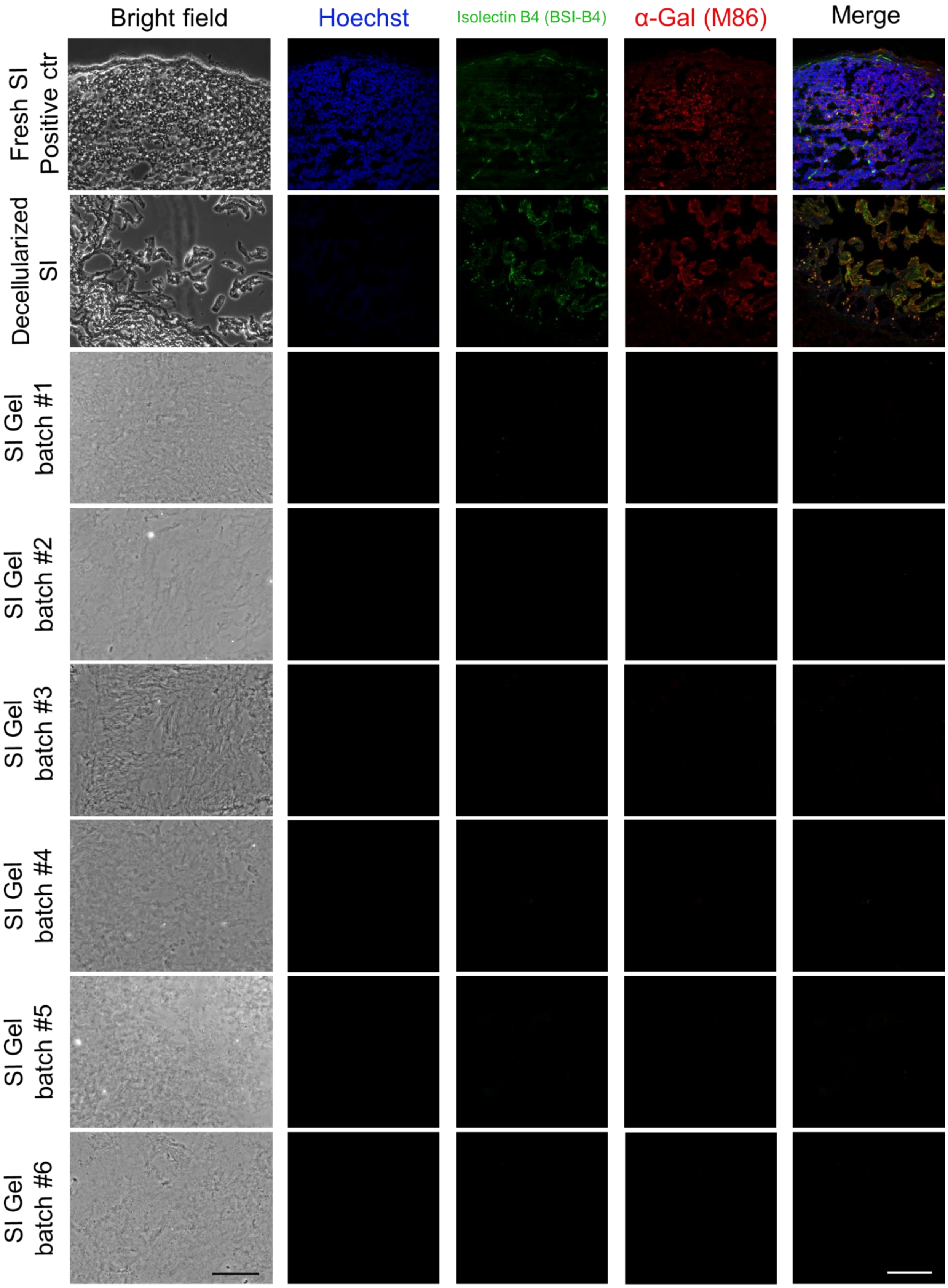

Supplementary Figure 4: Immunofluorescence staining of tissue and gel cryosections. Nuclei in blue, FITC-conjugated B4 isolectin (BSI-B4; Griffonia (Bandeiraea) simplicifolia) in green, and anti-alpha-Gal antibody (M86) in red. Immunofluorescence shows presence of alpha-gal antigen in fresh piglet small intestinal tissue, residual antigen presence in decellularized tissue, absence in 6 different batches of piglet ECM gels. Scale bars 100 µm.

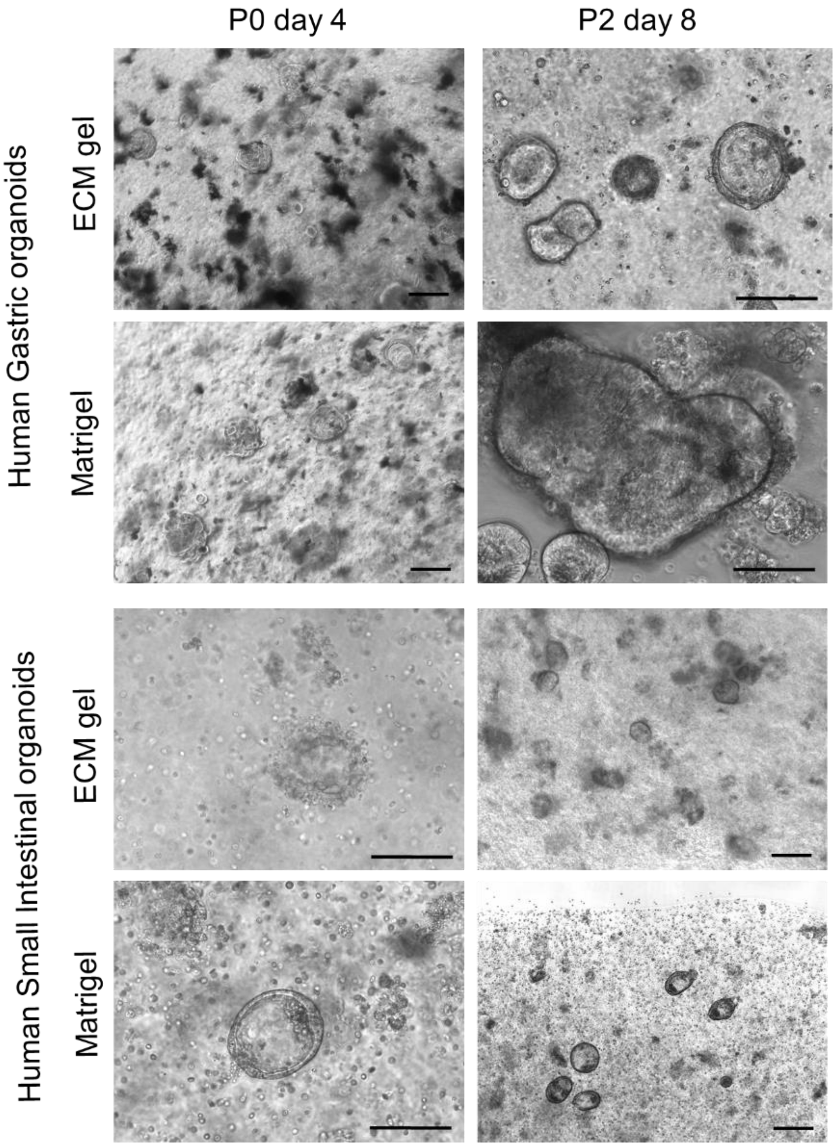

Supplementary Figure 5: Direct derivation of human gastric organoids, and human small intestinal organoids, from pediatric donor biopsies in 4 mg/mL small intestinal ECM gel and Matrigel control. Scale bars 200 µm.

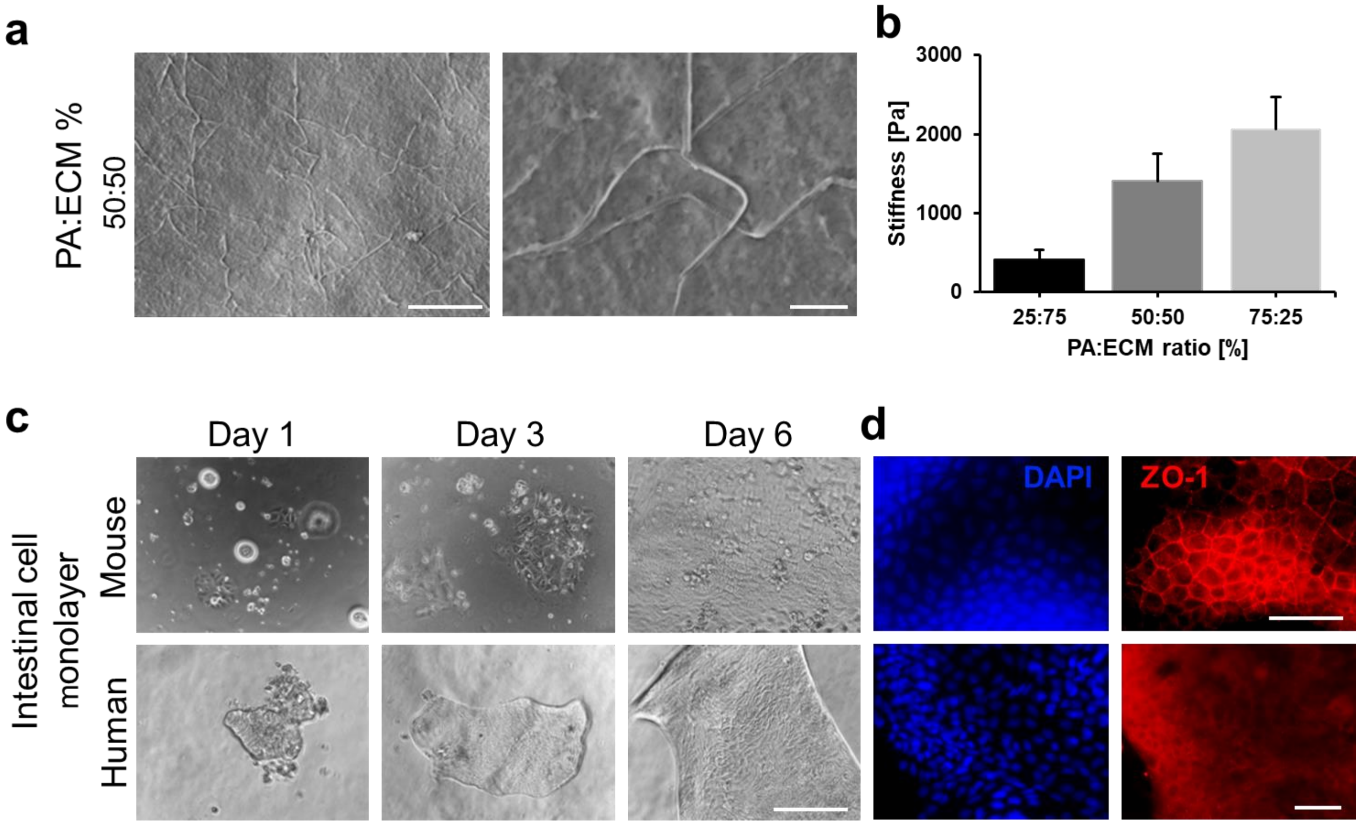

Supplementary Figure 6: Co-polymerization of the ECM-derived hydrogel with photo-crosslinkable polyacrylamide to design a flat hydrogel with tunable properties. (a) Scanning electron microscopy (SEM) images of the ECM-PA co-polymer hydrogel displaying the homogenous distribution of the ECM fibers on the surface of the polymer. Scale bars 10 µm and 1 µm. (b) Atomic Force Microscopy (AFM) characterization. This graph represents the Young's modulus of different hydrogel with growing concentration of polyacrylamide compared to ECM, showing the possibility to tune the stiffness properties of the co-polymer. (c) Mouse and human small intestinal organoids disaggregated to single cells and plated as monolayer on the ECM-PA hydrogel. The cells show adhesion and proliferation until confluence. Scale bar 100 µm. (d) Immunofluorescence staining showing epithelial colony organization. Scale bars 50 µm.

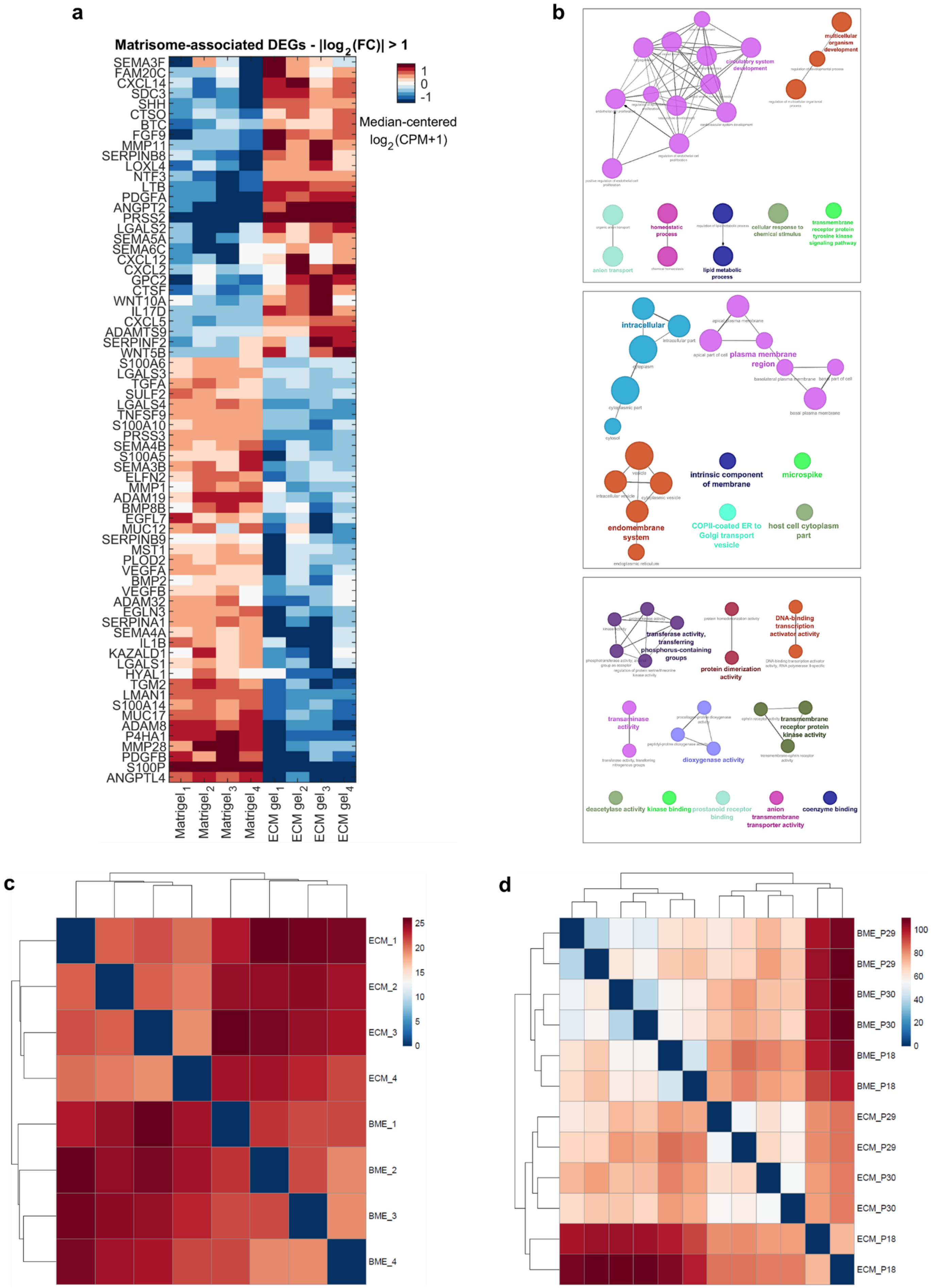
a
b
c
d
Matrisome-associated DEGs - $|log_2(FC)| > 1$
Median-centered
$log_2(CPM+1)$
1
0
-1
SEMA3F
FAM20C
CXCL14
SDC3
SHH
CTSO
BTC
FGF9
MMP11
SERPINB8
LOXL4
NTF3
LTB
PDGFA
ANGPT2
PRSS2
LGALS2
SEMA5A
SEMA6C
CXCL12
CXCL2
GPC2
CTSF
WNT10A
IL17D
CXCL5
ADAMTS9
SERPINF2
WNT5B
S100A6
LGALS3
TGFA
SULF2
LGALS4
TNFSF9
S100A10
PRSS3
SEMA4B
S100A5
SEMA3B
ELFN2
MMP1
ADAM19
BMP8B
EGFL7
MUC12
SERPINB9
MST1
PLOD2
VEGFA
BMP2
VEGFB
ADAM32
EGLN3
SERPINA1
SEMA4A
IL1B
KAZALD1
LGALS1
HYAL1
TGM2
LMAN1
S100A14
MUC17
ADAM8
P4HA1
MMP28
PDGFB
S100P
ANGPTL4
Matrigel 1
Matrigel 2
Matrigel 3
Matrigel 4
ECM gel 1
ECM gel 2
ECM gel 3
ECM gel 4
multicellular organism development
circulatory system development
homeostatic process
cellular response to chemical stimulus
transmembrane receptor protein tyrosine kinase signaling pathway
anion transport
lipid metabolic process
intracellular
plasma membrane region
intrinsic component of membrane
microspike
endomembrane system
COPII-coated ER to Golgi transport vesicle
host cell cytoplasm part
transferase activity, transferring phosphorus-containing groups
protein dimerization activity
DNA-binding transcription activator activity
transaminase activity
dioxygenase activity
transmembrane receptor protein kinase activity
deacetylase activity
kinase binding
prostanoid receptor binding
anion transmembrane transporter activity
coenzyme binding
ECM_1
ECM_2
ECM_3
ECM_4
BME_1
BME_2
BME_3
BME_4
25
20
15
10
5
0
BME_P29
BME_P29
BME_P30
BME_P30
BME_P18
BME_P18
ECM_P29
ECM_P29
ECM_P30
ECM_P30
ECM_P18
ECM_P18
100
80
60
40
20
0

Supplementary Figure 7. RNA-seq analysis of human organoids cultured in ECM gel vs Matrigel. (A-B) Results of human pediatric small intestinal organoids. (a) Hierarchical clustering of ECM-associated DEGs. (b) Results of over-representation analysis of DEGs within the following GO categories: GO-BP (top), GO-CC (middle), and GO-MF (bottom). Similar categories are clustered according to kappa score and indicated by different colors. Larger circle size indicates higher significance. Benjamini-Hochberg-corrected p-values from right-sided hypergeometric test. GO-BP corrected p-value<0.001, GO-CC and GO-MF corrected p-values< 0.05. (c) Cluster map of human ductal liver organoids cultured in ECM gel vs Matrigel. (d) Cluster map of human fetal hepatic organoids cultured in ECM gel vs Matrigel.

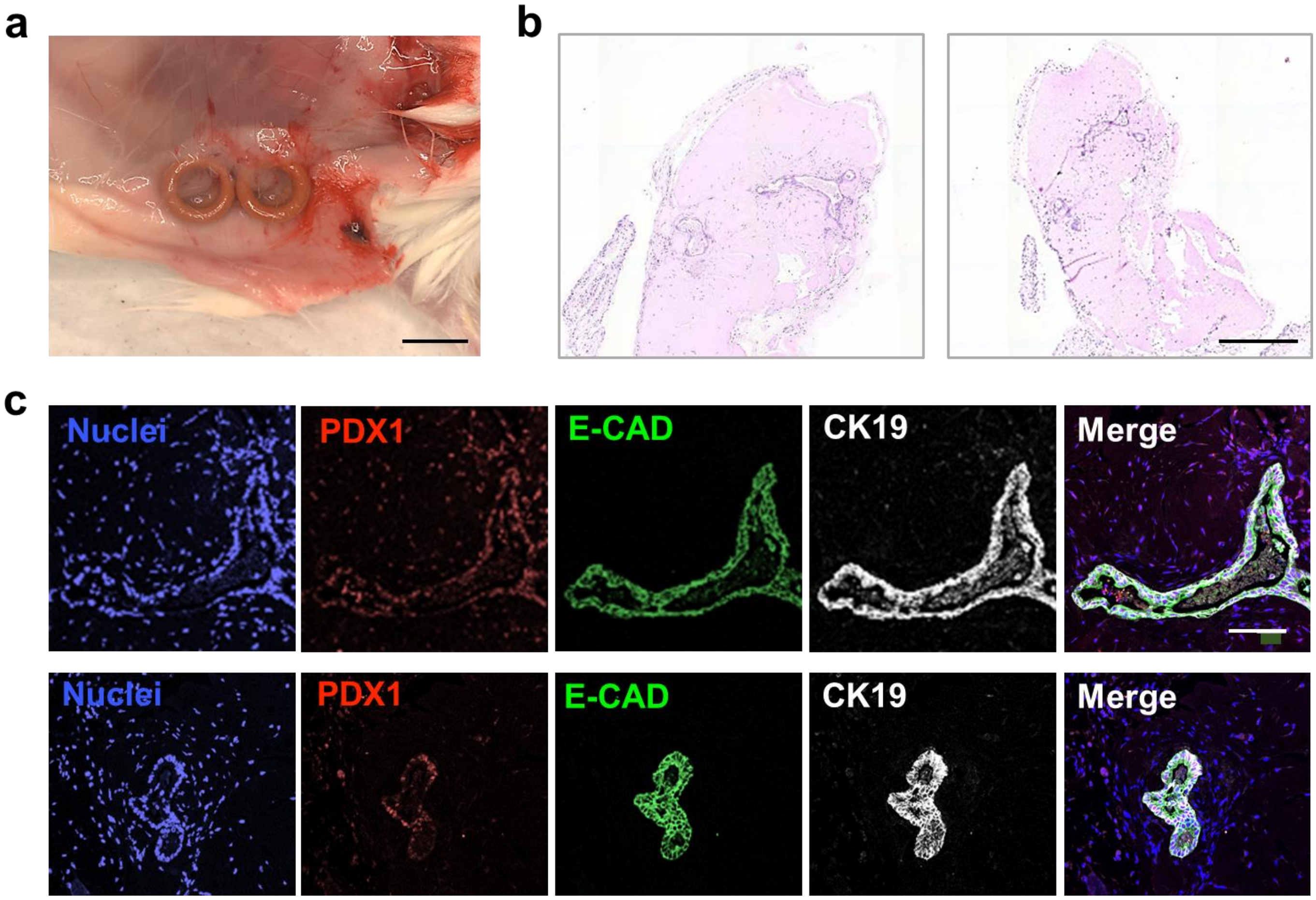

Supplementary Figure 8: Two months mouse subcutaneous transplantation of human fetal pancreatic organoids in ECM gels and Matrigel. (a) Recovery of silicon rings from mouse back with ECM gels after 2 months. Scale bar 5 mm. (b) H&E staining of pancreatic ducts showing comparable morphology after *in vivo* transplantation in ECM gel and control Matrigel. Scale bar 500 µm. (c) Immunofluorescence staining of human fetal ducts in ECM gel and Matrigel after 2 months *in vivo*, showing high expression of pancreatic markers insulin promoter factor 1, epithelial cadherin and cytokeratin-19. Scale bar 100 µm.

Supplementary Tables

Supplementary Table 1: Gelation characterization of different ECM concentrations

ECM gel	Lag Period (T_{lag}) (min, ±SD)	Gelation Rate (S) (AU/min, ±SD)	Gelation Half-time ($T_{1/2}$) (minutes, ±SD)
10mg/ml	16.39 ± 0.63	0.047 ± 6.33 x 10^{-4}	26.92 ± 0.78
8mg/ml	15.96 ± 0.82	0.048 ± 7.59 x 10^{-4}	26.30 ± 0.59
6mg/ml	12.79 ± 1.37 *	0.059 ± 2.66 x 10^{-3}	21.25 ± 0.83

Supplementary Table 2: Good Manufacturing Practice (GMP) marketed reagents

Good Manufacturing Practice (GMP) products	Company	Product code	Pack size
Milli-Q® Integral ultrapure water Type I, 0.22 µm filtered	Merck Millipore	ZRXQ015WW	unlimited
Sodium deoxycholate (SDC) PharmaGrade manufactured under appropriate controls	Merck - Sigma Aldrich	S1827	1 kg
Sodium chloride (NaCl) PharmaGrade, USP, Manufactured under appropriate GMP controls	Merck - Sigma Aldrich	RES0926S-A705X	25kg
DNase I, recombinant, RNase-free, GMP Grade	Roche - CustomBiotech	03724751103	4 kU
GMP-certified and virus validated Pepsin, pharmaceutical and technical grade	Biofac	Pepsin Powder – GMP grade	1 kg
Probumin® Bovine Serum Albumin (BSA) Biotech Grade	Merck - Sigma Aldrich	820476	5 kg
Hydrochloric acid (HCl) Grade ACS reagent, reag. ISO, reag. Ph. Eur. 37%	Merck - Sigma Aldrich	30721-1L-M	1 L

Supplementary Table 3: Mouse small intestinal organoid medium

Component	Stock conc.	Final conc.
Advanced DMEM F-12 (Thermo 12634)	-	To volume
HEPES (Thermo 15630080)	1 M	10 mM
Glutamax (Thermo 35050061)	100 X	2 mM
B-27 supplement minus vitamin A (Thermo 12587010)	50 X	1 X
n-acetylcysteine (Sigma A9165)	500 mM	1.25 mM
Pen/Strep (Thermo 15140122)	100 %	1 %
Wnt-3A (Peprotech 315-20) optional	50 µg/mL	100 ng/mL
R-spondin 1 (Peprotech 120-38)	100 µg/mL	500 ng/mL
Noggin (R&D 6057-NG)	100 µg/mL	100 ng/mL
EGF (Thermo PMG8043)	500 µg/mL	50 ng/mL

Supplementary Table 4: Human pediatric and fetal small intestinal organoid medium

Component	Stock conc.	Final conc.
Advanced DMEM F-12 (Thermo 12634)	-	To volume
HEPES (Thermo 15630080)	1 M	10 mM
Glutamax (Thermo 35050061)	100 X	2 mM
B-27 supplement minus vitamin A (Thermo 12587010)	50 X	1 X
n-acetylcysteine (Sigma A9165)	500 mM	1.25 mM
Pen/Strep (Thermo 15140122)	100 %	1 %
Wnt-3A (Peprotech 315-20)	50 µg/mL	100 ng/mL
R-spondin 1 (Peprotech 120-38)	100 µg/mL	500 ng/mL
Noggin (R&D 6057-NG)	100 µg/mL	100 ng/mL
EGF (Thermo PMG8043)	500 µg/mL	50 ng/mL
Gastrin (Sigma G9020)	100 µM	10 nM
GSK-3 inhibitor (CHIR 99021) (Tocris 4423)	3 mM	3 µM
TGFb inhibitor (A83-01) (Sigma SML0788)	500 µM	500 nM
P38 inhibitor (SB202190) (Sigma S7067)	30 mM	10 µM
Prostaglandin E2 (Cambridge cay14010)	100 µM	10 nM

Supplementary Table 5: Human pediatric gastric organoid medium

Component	Stock conc.	Final conc.
Advanced DMEM F-12 (Thermo 12634)	-	To volume
HEPES (Thermo 15630080)	1 M	10 mM
Glutamax (Thermo 35050061)	100 X	2 mM
B-27 supplement minus vitamin A (Thermo 12587010)	50 X	1 X
n-acetylcysteine (Sigma A9165)	500 mM	1.25 mM
Pen/Strep (Thermo 15140122)	100 %	1 %
Wnt-3A (Peprotech 315-20)	50 µg/mL	100 ng/mL
R-spondin 1 (Peprotech 120-38)	100 µg/mL	500 ng/mL
Noggin (R&D 6057-NG)	100 µg/mL	100 ng/mL
EGF (Thermo PMG8043)	500 µg/mL	50 ng/mL
Gastrin (Sigma G9020)	100 µM	10 nM
GSK-3 inhibitor (CHIR 99021) (Tocris 4423)	3 mM	3 µM
TGFb inhibitor (A83-01) (Sigma SML0788)	500 µM	5 µM
FGF10 (Peprotech 100-26)	100 µg/mL	200 ng/mL

Supplementary Table 6: Human fetal hepatocyte organoid medium

Component	Stock conc.	Final conc.
Advanced DMEM F-12 (Thermo 12634028)	-	To volume
Penicillin Streptomycin (Thermo 15140122)	100%	1%
L-Glutamine (Thermo 10378016)	100 X	1X
B-27 supplement (Gibco 17504-044)	50 X	1 X
R-spondin (conditioned medium Hans Clevers Lab)	10 mL	15%
EGF (Thermo PMG8043)	500 µg/mL	50 ng/mL
n-acetylcysteine (Sigma A9165)	500 mM	1.25 mM
Gastrin (Sigma G9020)	100 µM	10 nM
Nicotinamide (Sigma 72340)	1 M	10 mM
FGF7 (Peprotech 100-19)	50 µg/mL	100 ng/mL
FGF10 (Peprotech 100-26)	100 µg/mL	100 ng/mL
HGF (Peprotech 100-39)	20 µg/mL	50 ng/mL
GSK-3 inhibitor (CHIR 99021) (Tocris 4423)	3 mM	3 µM
TGFα (Peprotech 100-16A)	20 µg/mL	50 ng/mL
Primocin (Thermo)	50mg/mL	100 µg/mL

Supplementary Table 7: Human liver ductal organoid medium

Component	Stock conc.	Final conc.
Advanced DMEM F-12 (Thermo 12634028)	-	To volume
Penicillin Streptomycin (Thermo 15140122)	100%	1%
L-Glutamine (Thermo 10378016)	100 X	1X
B-27 supplement (Gibco 17504-044)	50 X	1 X
n-acetylcysteine (Sigma A9165)	500 mM	1.25 mM
R-spondin (conditioned medium Hans Clevers Lab)	10 mL	10%
EGF (Thermo PMG8043)	500 µg/mL	50 ng/mL
Gastrin (Sigma G9020)	100 µM	10 nM
FSK stock	10 µL	10 µM
FGF10 (Peprotech 100-26)	100 µg/mL	100 ng/mL
HGF (Peprotech 100-39)	20 µg/mL	25 ng/mL
TGFb inhibitor (A83-01) (Sigma SML0788)	5 mM	50 µM
Primocin (Thermo)	50mg/mL	100 µg/mL
Nicotinamide (Sigma 72340)	1 M	10 mM

Supplementary Table 8: Human fetal pancreatic organoid medium

Component	Stock conc.	Final conc.
Advanced DMEM F-12 (Thermo 12634028)	-	To volume
Penicillin Streptomycin (Thermo 15140122)	100%	1%
L-Glutamine (Thermo 10378016)	100 X	1X
B-27 supplement (Gibco 17504-044)	50 X	1 X
N-2 supplement (Gibco 17502-048)	50 X	1 X
Nicotinamide (Sigma 72340)	2 M	10 mM
N-Acetyl-L-cysteine (Sigma A9165)	1 mM	1 µM
R-spondin 1 (Peprotech 120-38)	100 µg/mL	500 ng/mL
Noggin (Peprotech 120-10C)	100 µg/mL	100 ng/mL
EGF (Peprotech AF-100-15)	50 µg/mL	50 ng/mL
FGF-10 (Peprotech AF-100-26)	100 µg/mL	100 ng/mL
Exendin4 (Sigma E7144)	100 µM	100 nM
Gastrin (Sigma G9020)	100 µM	100 nM

Supplementary Table 9: Antibody and molecule list

**Antibody/conjugated molecule**	**Dilution**
Ezrin (Thermo PA5-29358)	1:100
rFABP1 (R&D AF1565)	1:100
FITC-conjugated B4 isolectin (BSI-B4; Griffonia (Bandeiraea) simplicifolia) (Sigma L2895)	20 μg/mL
alpha-Gal (M86) (Enzo ALX-801-090-1)	1:5
GFP (Thermo A-21311)	1:100
E-cadherin (BD 610182)	1:100
Muc-1A (Termo HM-1630-P0)	1:200
PDX-1 (Abacm AB47308)	1:200
Sox-9 (Merk AB5535)	1:500
Ki-67 (ABCAM Ab15580)	1:200
Lysozyme (Genetex GTX72913)	1:100
Lysozyme (Genetex GTX39779)	1:100
Mucin-2 (Genetex GTX100664)	1:100
Villin (Genetex GTX109940)	1:100
Olfactomedin-4 (Cell signaling 14369S)	1:50
Cytokeratin-20 (Proteintech 60183-1-lg)	1:100
Zonula occludens-1 (Invitrogen 40-2200)	1:200
Mucin-5AC (Thermo MA5-12178)	1:100
Cytokeratin-19 (Abcam AB76539)	1:100
Phalloidin 488 (Thermo A12379)	1:200
Goat anti-Rabbit 594 (Thermo A11012)	1:500
Goat anti-Rabbit 568 (Thermo A11011)	1:500
Goat anti-Rabbit 488 (Thermo A11008)	1:500
Goat anti-Mouse 488 (Thermo A11001)	1:500
Goat anti-Mouse 568 (Thermo A10037)	1:500
Donkey anti-Goat 647 (Thermo A-21447)	1:500
Anti-Guinea pig (Jackson 706-165-148)	1:500
Anti-Hamster (Abcam AB175716)	1:500
Hoechst 33342 (Thermo H1399)	10 μg/mL
Calcein-AM (Thermo L3224)	3 μM
Ethidium homodimer-1 (Thermo L3224)	3 μM
