# Supplementary material for: Extracellular matrix hydrogel derived from decellularized tissues enables endodermal organoid culture
Source: Nat Commun. 2019 Dec 11;10:5658. doi: 10.1038/s41467-019-13605-4 (PMC6906306; doi:10.1038/s41467-019-13605-4)
Supplement: Supplementary file 2 — Reporting Summary [file 41467_2019_13605_MOESM2_ESM.pdf]

nature research

Corresponding author(s): Paolo De Coppi, Nicola Elvassore

Last updated by author(s): Oct 7, 2019

Reporting Summary

Nature Research wishes to improve the reproducibility of the work that we publish. This form provides structure for consistency and transparency in reporting. For further information on Nature Research policies, see Authors & Referees and the Editorial Policy Checklist.

Statistics

For all statistical analyses, confirm that the following items are present in the figure legend, table legend, main text, or Methods section.

n/a	Confirmed	
☐	☒	The exact sample size (*n*) for each experimental group/condition, given as a discrete number and unit of measurement
☐	☒	A statement on whether measurements were taken from distinct samples or whether the same sample was measured repeatedly
☐	☒	The statistical test(s) used AND whether they are one- or two-sided<br>*Only common tests should be described solely by name; describe more complex techniques in the Methods section.*
☒	☐	A description of all covariates tested
☒	☐	A description of any assumptions or corrections, such as tests of normality and adjustment for multiple comparisons
☐	☒	A full description of the statistical parameters including central tendency (e.g. means) or other basic estimates (e.g. regression coefficient) AND variation (e.g. standard deviation) or associated estimates of uncertainty (e.g. confidence intervals)
☐	☒	For null hypothesis testing, the test statistic (e.g. *F*, *t*, *r*) with confidence intervals, effect sizes, degrees of freedom and *P* value noted<br>*Give P values as exact values whenever suitable.*
☒	☐	For Bayesian analysis, information on the choice of priors and Markov chain Monte Carlo settings
☐	☒	For hierarchical and complex designs, identification of the appropriate level for tests and full reporting of outcomes
☒	☐	Estimates of effect sizes (e.g. Cohen's *d*, Pearson's *r*), indicating how they were calculated

Our web collection on statistics for biologists contains articles on many of the points above.

Software and code

Policy information about availability of computer code

Data collection	For human pediatric small intestinal organoids, Illumina novaSeq base call (BCL) files were converted into fastq files through bcl2fastq (version v2.20.0.422) following software guide. Sequence reads were trimmed using bbduk software (bbmap suite 37.31), following software guide, to remove adapter sequences, poly-A tails and low-quality end bases (regions with average quality below 6). Alignment was performed with STAR 2.6.0a47 on hg38 reference assembly obtained from cellRanger website (Ensembl 93), following online site guide. The expression levels of genes were determined with htseq-count 0.9.1 by using cellRanger pre-build genes annotations (Ensembl Assembly 93).
Data analysis	For Student-t test, Prism 6 was used.<br>For RNA Seq, all genes having <1 CPM in less than 4 samples and percentage of multimap alignment reads > 20% simultaneously were filtered out. Differentially expressed genes (DEGs) were computed with edgeR, using a mixed criterion based on p-value, after false discovery rate (FDR) correction by Benjamini-Hochberg method, lower than 0.05 and absolute log2(fold change) higher than 1. A Principal Component Analysis was performed by Singular Value Decomposition (SVD) on log2(CPM+1) data, after centering, using MATLAB R2019a (The MathWorks). Hierarchical clustering of ECM-related gene sets was performed with Euclidean distance and complete linkage using median-centered data, and plotted as heat maps using MATLAB. DEGs over-representation analysis of Gene Ontology (GO) categories was performed using ClueGO (version 2.5.4).

For manuscripts utilizing custom algorithms or software that are central to the research but not yet described in published literature, software must be made available to editors/reviewers. We strongly encourage code deposition in a community repository (e.g. GitHub). See the Nature Research guidelines for submitting code & software for further information.

October 2018

Data

Policy information about availability of data

All manuscripts must include a data availability statement. This statement should provide the following information, where applicable:

- Accession codes, unique identifiers, or web links for publicly available datasets
- A list of figures that have associated raw data
- A description of any restrictions on data availability

The authors declare that all data supporting the findings of this study are available within the article, its Supplementary Information, attached files, and online links, or from the authors upon reasonable request.

Field-specific reporting

Please select the one below that is the best fit for your research. If you are not sure, read the appropriate sections before making your selection.

☒ Life sciences ☐ Behavioural & social sciences ☐ Ecological, evolutionary & environmental sciences

For a reference copy of the document with all sections, see nature.com/documents/nr-reporting-summary-flat.pdf

Life sciences study design

All studies must disclose on these points even when the disclosure is negative.

Sample size	Sample size varied from 3 to 30 depending on the type of measure to be conducted.
Data exclusions	No data were excluded from the analyses
Replication	Experiments were replicated within our laboratory, and independently across laboratories and Universities.
Randomization	Not relevant
Blinding	For organoids diameter counts, and colony formation counting, the people in charge were blinded and were not previously informed of the samples and control they were analyzing.

Reporting for specific materials, systems and methods

We require information from authors about some types of materials, experimental systems and methods used in many studies. Here, indicate whether each material, system or method listed is relevant to your study. If you are not sure if a list item applies to your research, read the appropriate section before selecting a response.

Materials & experimental systems

n/a	Involved in the study	
☐	☒	Antibodies
☒	☐	Eukaryotic cell lines
☒	☐	Palaeontology
☐	☒	Animals and other organisms
☒	☐	Human research participants
☒	☐	Clinical data

Methods

n/a	Involved in the study	
☒	☐	ChIP-seq
☒	☐	Flow cytometry
☒	☐	MRI-based neuroimaging

Antibodies

Antibodies used

Ezrin (Thermo PA5-29358) 1:100
rFABP1 (R&D AF1565) 1:100
FITC-conjugated B4 isolectin (BSI-B4; Griffonia (Bandeiraea) simplicifolia) (Sigma L2895) 20 µg/mL
alpha-Gal (M86) (Enzo ALX-801-090-1) 1:5
GFP (Thermo A-21311) 1:100
E-cadherin (BD 610182) 1:100
Muc-1A (Termo HM-1630-P0) 1:200
PDX-1 (Abacm AB47308) 1:200
Sox-9 (Merk AB5535) 1:500
Ki-67 (ABCAM Ab15580) 1:200
Lysozyme (Genetex GTX72913) 1:100
Lysozyme (Genetex GTX39779) 1:100
Mucin-2 (Genetex GTX100664) 1:100

Villin (Genetex GTX109940) 1:100
Olfactomedin-4 (Cell signaling 14369S) 1:50
Cytokeratin-20 (Proteintech 60183-1-lg) 1:100
Zonula occludens-1 (Invitrogen 40-2200) 1:200
Mucin-5AC (Thermo MA5-12178) 1:100
Cytokeratin-19 (Abcam AB76539) 1:100
Phalloidin 488 (Thermo A12379) 1:200
Goat anti-Rabbit 594 (Thermo A11012) 1:500
Goat anti-Rabbit 568 (Thermo A11011) 1:500
Goat anti-Rabbit 488 (Thermo A11008) 1:500
Goat anti-Mouse 488 (Thermo A11001) 1:500
Goat anti-Mouse 568 (Thermo A10037) 1:500
Donkey anti-Goat 647 (Thermo A-21447) 1:500
Anti-Guinea pig (Jackson 706-165-148) 1:500
Anti-Hamster (Abcam AB175716) 1:500
Hoechst 33342 (Thermo H1399) 10 µg/mL
Calcein-AM (Thermo L3224) 3 µM
Ethidium homodimer-1 (Thermo L3224) 3 µM

Validation

Validated through positive and negative controls, and following supplier information.

Animals and other organisms

Policy information about studies involving animals; ARRIVE guidelines recommended for reporting animal research

Laboratory animals	CD1 mice, LGR5-DTR-EGFP mice, NODSCID-gamma (NSG) mice (all females)
Wild animals	No wild animals used
Field-collected samples	No samples collected in the fields
Ethics oversight	Animal work was ethically approved and carried out under Home Office Project Licence PPL PDD3A088A.

Note that full information on the approval of the study protocol must also be provided in the manuscript.
