# Supplementary material for: Extracellular matrix hydrogel derived from decellularized tissues enables endodermal organoid culture
Source: Nat Commun. 2019 Dec 11;10:5658. doi: 10.1038/s41467-019-13605-4 (PMC6906306; doi:10.1038/s41467-019-13605-4)
Supplement: Supplementary file 3 — Description of Additional Supplementary Files [file 41467_2019_13605_MOESM3_ESM.pdf]

Description of Additional Supplementary Files

File Name: Supplementary Data 1

Description: Small intestinal ECM powder proteomic analysis. This file contains the pre-analysed information derived from the shotgun proteomic analysis, used to prepare the figures reported in the Main Figure 2 on the manuscript.

File Name: Supplementary Data 2

Description: Small intestinal organoid transcriptomic analysis. This file contains the DEGs identified by 3' RNA sequencing of the human small intestinal organoids cultured in ECM gel VS Matrigel, and the GOBP, GOCC, and GOMF from the biological processes Uniprot analysis
